# Tudor-SN exacerbates pathological vascular remodeling by promoting the polyubiquitination of PTEN via NEDD4-1

**DOI:** 10.1186/s12929-024-01076-9

**Published:** 2024-09-05

**Authors:** Yichen Wu, Zilong Chen, Zhe Zheng, Xiaoguang Li, Jiangcheng Shu, Ruiqi Mao, Jie An, Siyuan Fan, Ruijie Luo, Yi Guo, Wenjing Xu, Minglu Liang, Kai Huang, Cheng Wang

**Affiliations:** 1 Department of Cardiology, Union Hospital, Tongji Medical College, Huazhong University of Science and Technology, 1277 Jiefang Ave, Wuhan, 430022 Hubei China; 2 Clinic Center of Human Gene Research, Union Hospital, Tongji Medical College, Huazhong University of Science and Technology, Wuhan, 430022 Hubei China; 3 Department of Rheumatology, Union Hospital, Tongji Medical College, Huazhong University of Science and Technology, 1277 Jiefang Ave, Wuhan, 430022 Hubei China; 4 Department of Cardiology, The First Affiliated Hospital of Zhengzhou University, Zhengzhou, 450052 Henan China; 5 Hubei Key Laboratory of Metabolic Abnormalities and Vascular Aging, Wuhan, China; 6 Hubei Clinical Research Center for Metabolic and Cardiovascular Disease, Wuhan, China

**Keywords:** Vascular remodeling, Polyubiquitination, Smooth muscle cells, Tudor-SN

## Abstract

**Background:**

Dysregulation of vascular homeostasis can induce cardiovascular diseases and increase global mortality rates. Although lineage tracing studies have confirmed the pivotal role of modulated vascular smooth muscle cells (VSMCs) in the progression of pathological vascular remodeling, the underlying mechanisms are still unclear.

**Methods:**

The expression of Tudor-SN was determined in VSMCs of artery stenosis, PDGF-BB-treated VSMCs and atherosclerotic plaque. Loss- and gain-of-function approaches were used to explore the role of Tudor-SN in the modulation of VSMCs phenotype both in vivo and in vitro.

**Results:**

In this study, we demonstrate that Tudor-SN expression is significantly elevated in injury-induced arteries, atherosclerotic plaques, and PDGF-BB-stimulated VSMCs. Tudor-SN deficiency attenuates, but overexpression aggravates the synthetic phenotypic switching of VSMCs and pathological vascular remodeling. Loss of Tudor-SN also reduces atherosclerotic plaque formation and increases plaque stability. Mechanistically, PTEN, the major regulator of the MAPK and PI3K-AKT signaling pathways, plays a vital role in Tudor-SN-mediated regulation on proliferation and migration of VSMCs. Tudor-SN facilitates the polyubiquitination and degradation of PTEN via NEDD4-1, thus exacerbating vascular remodeling under pathological conditions. BpV (HOpic), a specific inhibitor of PTEN, not only counteracts the protective effect of Tudor-SN deficiency on proliferation and migration of VSMCs, but also abrogates the negative effect of carotid artery injury-induced vascular remodeling in mice.

**Conclusions:**

Our findings reveal that Tudor-SN deficiency significantly ameliorated pathological vascular remodeling by reducing NEDD4-1-dependent PTEN polyubiquitination, suggesting that Tudor-SN may be a novel target for preventing vascular diseases.

**Supplementary Information:**

The online version contains supplementary material available at 10.1186/s12929-024-01076-9.

## Introduction

Vascular smooth muscle cells (VSMCs) play crucial roles in maintaining the structural integrity of blood vessels and regulating blood pressure [1, 2]. These cells are also characterized by their unique ability to adapt their cell phenotype under various physiological and pathological conditions. Extensive researches have confirmed the aberrant expansion of VSMCs in the progression of atherosclerosis, aortic aneurysm, pulmonary arterial hypertension, and restenosis after vascular injury, highlighting the importance of regulating their cellular fate [3–8]. In response to vascular injury, VSMCs undergo a phenotypic transition from a quiescent contractile state to an active synthetic state, causing subsequent arterial stenosis and impacting the prognosis of percutaneous coronary intervention (PCI) [6–8]. During the progression of atherosclerosis, which encompasses endothelial injury, lipid accumulation, cell proliferation/ migration, leukocyte infiltration, cell death and thrombosis, the regulation of VSMCs’ cellular fate exerts great impact on intimal thickening and plaque stability [9–11]. Therefore, unraveling these underlying mechanisms holds great significance in providing effective prevention and treatment strategies for in-stent restenosis and atherosclerosis.

Staphylococcal nuclease and Tudor domain-containing 1 (Tudor-SN, TSN) is a highly conserved protein with diverse biological functions [12]. Accumulating evidence strongly supports the essential role of Tudor-SN in promoting the progression of multiple human cancers [13–16]. However, despite the reported involvement of Tudor-SN in cell transcriptional regulation, RNA interference, mRNA splicing and stabilization, its potential contribution to vascular remodeling remains unclear. In this study, we revealed the strong involvement of Tudor-SN in the control of pathological vascular remodeling by orchestrating the cellular fate of VSMCs. Mechanistically, Tudor-SN was found to regulate the degradation of PTEN, a well-known tumor suppressor protein that also has critical functions in cardiovascular diseases, via NEDD4-1, an established E3 ubiquitin ligase for PTEN. Thus, targeting Tudor-SN may be a novel approach for preventing aberrant vascular remodeling.

## Methods

### Analysis of human arteries

To examine the expression pattern of Tudor-SN in healthy human coronary arteries, we utilized RNA-seq data of 240 human samples from the Genotype-Tissue Expression (GTEx) project [17]. Meanwhile, diseased human artery samples were analyzed using data from the Gene Expression Omnibus (GEO) database.

### Animal experiments, anesthesia and euthanasia

SMMHC-CreERT2 mice (SMMHC-Cre mice, purchased from Wuhan Youdu Biotechnology) were bred with mice carrying floxed alleles for the Tudor-SN gene (TSN^fl/fl^ mice, purchased from Viewsolid Biotech) to generate TSN^fl/fl^; SMMHC-Cre mice and with mice carrying the human Tudor-SN CDS (hTSN.Stop^fl/−^ mice) to generate the hTSN.Stop^fl/−^; SMMHC-Cre mice. Tamoxifen (2 mg for 5 consecutive days) was injected into male mice to induce specific deletion or overexpression of Tudor-SN in VSMCs (abbreviated as TSN^smcKO^ or hTSN^smcKI^ mice, respectively). Male littermates with the TSN^WT/WT^; SMMHC-Cre and hTSN^WT/WT^; SMMHC-Cre genotypes were used as the control groups for validation (abbreviated as TSN^WT^ and hTSN^WT^ mice, respectively). To obtain atherosclerotic models, TSN^fl/fl^;SMMHC-Cre mice were crossed with Apoe^−/−^ mice (B6/JGpt-*Apoe*^em1Cd82^/Gpt, purchased from GemPharmatech Co.Ltd) to generate TSN^fl/+^; SMMHC-Cre; Apoe^−/−^ males and TSN^fl/+^; SMMHC-Cre; Apoe^−/−^ females, which were then used as breeders to generate male experimental and control littermate mice. All mice were housed in a specific pathogen-free (SPF)-grade facility at Huazhong University of Science and Technology and maintained on a 12 h light/dark cycle with free access to food and water. The identification of mouse genotypes was conducted using polymerase chain reaction (PCR). All animal experiments were performed with the permission of the Animal Experimentation Committee of Huazhong University of Science and Technology (IACUC Number: 3416), which were conducted according to the Animals (Scientific Procedures) Act of 1986 (United Kingdom) and conformed to the guidelines from Directive 2010/63/EU of the European Parliament on the protection of animals used for scientific purposes or the NIH guidelines (Guide for the care and use of laboratory animals). For mouse carotid artery ligation, anesthesia was induced using 100% O_2_/4% isoflurane, and was maintained throughout the procedure by the administration of 100% O_2_/2% isoflurane. At the end of protocol, all mice were euthanized by placing them under deep anesthesia with 100% O_2_/5% isoflurane, followed by decapitation.

### Establishment of the CAL model

Eight-week-old anesthetized male C57BL/6 J mice were subjected to carotid artery ligation (CAL) surgery as previously described [6] and sacrificed for tissue section staining and Western blot analysis at four time points after surgery (n ≥ 6). Tamoxifen-induced TSN^WT^/ TSN^smcKO^/ hTSN^WT^/ hTSN^smcKI^ mice were sacrificed 14 days post-surgery (n = 6). Body weight was measured every other day after ligation.

### Establishment of the atherosclerotic model

Eight-week-old mice were fed with a high-fat diet (D12108C; Medicience, Jiangsu, China) for 16 weeks. Anesthetized mice were sacrificed to collect the blood plasma, hearts and aortas. The hearts were molded in optimal cutting temperature (OCT) compounds for cryosectioning of the aortic sinus after Oil Red O and collagen staining. The aortas were opened longitudinally for Oil Red O staining and en-face analysis.

### Histomorphometry

Vascular tissue sections were fixed in 4% paraformaldehyde and embedded in paraffin by Bios Biological Company. Sections were subsequently stained with hematoxylin and eosin (H&E) to visualize vascular remodeling. Masson staining was conducted to observe the collagen fibers. Immunohistochemistry of Tudor-SN within the atherosclerotic plaque was performed to demonstrate expression change (Abclonal, A5874, 1:800 dilution). Images were taken using an inverted microscope (Olympus IX73). ImageJ software was used to calculate the area of the neointima, vessel media and vessel lumen to assess the degree of neointimal hyperplasia.

### Immunofluorescence

Vascular tissue sections were dewaxed, boiled for antigen retrieval in citrate buffer, and blocked in 5% goat serum (dissolved in PBS) for 1 h at room temperature. Primary antibodies targeting Tudor-SN (Abclonal, A5874, 1:100 dilution), α-SMA (Abclonal, A17910, 1:100 dilution), Cyclin D1 (Proteintech, 60186-1-Ig, 1:100 dilution) and MMP9 (Proteintech, 10375-2-AP, 1:100 dilution) were applied to the slides in a humidified chamber at 4℃ overnight. The next day, the slides were incubated with secondary antibodies (Alexa Fluor 488- and Alexa Fluor 555-conjugated) for 1 h at room temperature. DAPI (Servicebio, G1012) was then added to the slides for 15 min at room temperature for nuclear staining. All immunofluorescence micrographs were captured using a confocal microscope (Nikon). The mean fluorescence intensity and positive area ratio were analyzed using ImageJ software.

### SMC isolation and culture

For primary mouse SMC isolation, aortas were first obtained from 4 to 6 mice with the adipose tissue removed delicately and then digested in solution containing collagenase (0.375 mg/ml, Worthington) and elastase (0.1 mg/ml, Worthington) at 37 ℃ for 10 min. The adventitial tissue was carefully stripped off with microscopic tweezers, and the endothelial cells were gently wiped off with cotton. The remaining aortas were washed 3 times with D-hanks solution and cultured at 37 ℃ in a 5% CO_2_ incubator in DMEM supplemented with 10% FBS (Gibco). The next day, the aortas were cut into small pieces using microscissors and digested again with a solution consisting of collagenase (1.375 mg/ml) and elastase (0.01 mg/ml) at 37 ℃ for 1 h. The cells were then centrifuged, resuspended and cultured in DMEM supplemented with 10% FBS. All VSMCs used in the experiments were between the third and eighth passages.

### Protein extraction and western blot analysis

For aortic protein extraction, the adipose tissue, adventitia and endothelial cells were removed as described above. The remaining tissues were ground with glass grinders with prepared RIPA lysis buffer (Servicebio, G2002) containing PMSF (Servicebio, G2008) and phosphatase inhibitor (Servicebio, G2007) until they were dissolved. For carotid arteries, only the adipose tissue was removed. The tissues were subsequently lysed with an ultrasonic disperser on ice, and the protein concentration was determined with a Pierce^™^ BCA protein assay kit (Thermo Scientific^™^, 23227). Proteins subjected to different treatments were separated by sodium dodecyl sulfate‒polyacrylamide gel electrophoresis (SDS‒PAGE), transferred to PVDF membranes (Millipore, IPVH00010), and later blocked with 5% skim milk. Primary antibodies targeting Tudor-SN (Abclonal, A5874, 1:1000 dilution), Cyclin D1 (Proteintech, 60186–1-Ig, 1:1000 dilution), SM22 (Proteintech, 10493-1-AP, 1:1000 dilution) and GAPDH (Proteintech, 60004–1-Ig, 1:1000 dilution) were subsequently applied to the blots at 4℃ overnight. The next day, the blots were incubated with HRP-conjugated secondary antibodies (Proteintech, SA00001-1, SA00001-2, 1:10000 dilution), and the target bands were visualized using BeyoECL Moon (Beyotime, P0018FS). Cell protein extraction and detection were conducted as previously described[6] using primary antibodies targeting GAPDH (Proteintech, 60004-1-Ig, 1:1000 dilution), α-Tubulin (Proteintech, 11224-1-AP, 1:1000 dilution), p-ERK1/2 (CST, #4370, 1:1000 dilution), ERK1/2 (CST, #4695, 1:1000 dilution), p-JNK (CST, #4668, 1:1000 dilution), JNK (Bimake, A5005, 1:500 dilution), p-P38 (CST, #9216, 1:500 dilution), P38 (CST, #8690, 1:1000 dilution), p-c-JUN (Abclonal, AP0105, 1:1000 dilution), c-JUN (CST, #9165, 1:1000 dilution), p-AKT (CST, #4060, 1:1000 dilution), AKT (CST, #4691, 1:1000 dilution), p-P13K (CST, #17366, 1:1000 dilution), PI3K (CST, #11889), PTEN (Abclonal, A19104, 1:1000 dilution), P53 (Abclonal, A0263, 1:1000 dilution), CREBBP (Abclonal, A25323, 1:1000 dilution), Flag (Sigma-Aldrich, F1804, 1:500 dilution), NEDD4-1 (Proteintech, 21698-1-AP, 1:500 dilution) and Ub (Proteintech, 10201-2-AP, 1:500 dilution). ImageJ software was used for all densitometric analyses.

### Cell proliferation assay

For evaluation of cell proliferation, MASMCs isolated from TSN^WT^, TSN^smcKO^ and wild-type mice pretreated with Ad-Null/Ad-TSN were seeded onto different 96-well plates (10,000/well) and cultured to near confluence. The cells were then starved in FBS-free DMEM for 24 h and treated with vehicle or 20 ng/ml PDGF-BB (GenScript, Z03572). CCK-8 and EdU assays were conducted as previously described [6]. ImageJ software was used for the analyses.

### Cell migration assay

For analysis of the cell migratory ability, the above MASMCs were seeded onto 6-well plates for wound healing assays and into the upper transwell chambers of 24-well plates for transwell assays. The experiments were carried out as previously described [6]. ImageJ software was used for the analyses.

### Collagen gel contraction assay

The Cell Contraction Assay Kit (CBA-201, purchased from Cell Biolabs, Inc.) was used to assess cell contractile ability. Experiments were performed following the manufacturer’s instructions, and the photos of the wells were taken 24 h after initiating contraction. ImageJ software was used for gel area analysis.

### RNA sequencing

Aortas from TSN^WT^ and TSN^smcKO^ mice were obtained and digested as described above. After the adventitia was removed, the vessels were swiftly rinsed with prechilled PBS and placed in a 1.5 ml EP tube for rapid freezing in liquid nitrogen. The frozen tissues can be stored at −80 ℃ or promptly subjected to experiments. Subsequent RNA sequencing was conducted by Wuhan SeqHealth Technology.

### Plasmid construction and transfection

The wild-type and mutant forms of hTudor-SN were constructed and validated by Hanbio Tech (Shanghai, China). The plasmids used in luciferase reporter assay were purchased from Addgene (Watertown, MA) or MiaolingBio (Wuhan, China). When the HEK293T cells reached approximately 80% confluence, the medium was replaced with serum-free DMEM. Transfection solution was prepared using RNATransMate (Sangon Biotech, E607402).

### Coimmunoprecipitation assay

Cell protein extraction was conducted as described above. The protein samples were incubated with specific antibodies or IgG (CST, #3900, #5415) at 4 ℃ overnight, and protein A/G magnetic beads (MCE, HY-K0202) were added for another incubation on a rotator for 2 h. The samples were then centrifuged and washed with wash buffer (150 mmol/L NaCl, 20 mmol/L Tris–HCl, pH 7.5, 5% glycerol, 1 mmol/L MgCl_2_, and 1 mmol/L EDTA) 6 times before being boiled for subsequent immunoblotting.

### RNA extraction and RT‒PCR

Cell RNA was extracted using TRIzol reagent (Invitrogen, 15596018) and reverse transcribed (PrimeScript RT Master Mix Kit, TaKaRa, RR036A). Real-time polymerase chain reactions (RT‒PCRs) were subsequently performed with SYBR Premix Ex Taq (TaKaRa, RR420A) and an ABI 7500 Real-Time PCR system (Thermo Fisher Scientific). Relative fold changes in the expression of mRNAs of interest were normalized to that of the 18S gene and calculated with the 2^−ΔΔCT^ method. All primers used for RT‒PCR are listed in Supplementary Table S1.

### Luciferase reporter assay

The 2-kp promoter for the coding sequence of the human Tudor-SN gene was subcloned into the pGL3 luciferase reporter vector (Promega, USA). Luciferase reporter constructs were co-transfected with an internal control plasmid pRL-TK (Renilla luciferase reporter plasmid, Promega) into HEK293T cells. The Dual Luciferase Reporter Assay Kit (TransGen Biotech, FR201-02-V2) was used for examination according to the manufacturer’s instructions. Primers used to amplify human Tudor-SN promoter are listed in Supplementary Table S1.

### Statistical analysis

All the statistical analyses were performed using GraphPad Prism 8, and all the values are presented as the means ± standard deviations. The normality of the data was first tested using the Shapiro‒Wilk test. For normally distributed data, statistical analyses between two groups were conducted using standard Student’s t tests; one-/two-way ANOVA with a post hoc test of Tukey’s analysis was used for analyses of data from more than two groups. At least 3 independent experiments were performed, and *P < 0.05 were considered to indicate statistical significance.

## Results

### Tudor-SN is upregulated during vascular remodeling

**Fig. 1:**
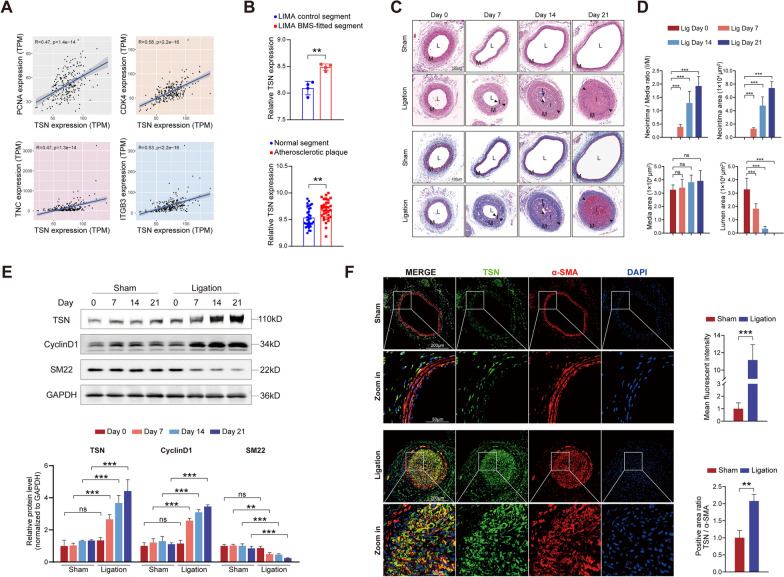
Tudor-SN is involved in pathological vascular remodeling. **A** RNA-seq data of human normal coronary artery specimens from the GTEx database were subjected to correlation analysis between Tudor-SN and genes associated with vascular remodeling (n = 240). **B** The gene expression profiles of human LIMA specimens (n = 4, up) and atherosclerotic artery specimens (n = 32, bottom) from the GEO database were collected and analyzed for Tudor-SN expression. **C** H&E and Masson staining of sham-operated and ligated artery sections obtained at different time intervals postsurgery (n = 6). L: lumen, indicated by blue arrowheads in the third column; M: media; I: neointima, highlighted by black arrowheads. Scale bar: 100 μm. **D** Statistical analyses of the I/M ratio, neointimal area, media area, and lumen area of the ligated arteries. **E** Western blot analysis of Tudor-SN, CyclinD1, and SM22 in sham-operated and ligated arteries at different time intervals postsurgery (n = 6). Statistical analyses of the relative expression levels are shown below. **F** Double immunofluorescence staining of Tudor-SN (green) and α-SMA (red) in sham-operated and ligated artery sections 14 days postsurgery (n = 3). Nuclei were stained with DAPI (blue). Scale bar: 200 μm and 50 μm (zoom in). Statistical analyses of Tudor-SN mean fluorescence intensity and positive area ratio of Tudor-SN/α-SMA (both normalized to the sham group) are shown on the right. Throughout, the results are presented as the mean ± SD; statistical analyses were conducted using Student’s t tests in data B & F and one-way ANOVA with a post hoc test of Tukey’s analysis in data **D** & **E**; *p < 0.05, **p < 0.01, ***p < 0.001; *ns* nonsignificant

We initially investigated the expression pattern of Tudor-SN in human arterial tissues. By analyzing the GTEx database, we discovered a positive correlation between the expression of Tudor-SN and several genes associated with VSMCs proliferation and migration (PCNA, CDK4, TNC, and ITGB3) in normal coronary artery specimens from humans (Fig. 1A). The gene expression profiles were subsequently retrieved from the Gene Expression Omnibus (GEO) database for further analysis. In the GSE19136 dataset, Tudor-SN expression was significantly greater in the BMS-implanted left internal mammary artery (LIMA) segments than in the normal LIMA segments (Fig. 1B). The GSE43292 dataset was also examined to evaluate the potential involvement of Tudor-SN in atherosclerosis progression, revealing an increase in Tudor-SN expression in human atherosclerotic plaques (Fig. 1B). Collectively, these findings indicate that Tudor-SN may play a crucial role in human vascular remodeling diseases. Next, we evaluated the expression of Tudor-SN during vascular intimal hyperplasia using a mouse carotid artery ligation (CAL) model. Carotid artery samples were collected bilaterally at different time intervals after surgery (0/7/14/21 days). H&E staining demonstrated the occurrence of intimal hyperplasia, while Masson staining provided enhanced visualization of collagen fiber secretion patterns (Fig. 1C). The ratio of the neointima area to the media area progressively increased over time, and this change was accompanied by a gradual reduction in the lumen area (Fig. 1D). In contrast, vessels from the sham-operated group exhibited vasodilatory remodeling due to compensatory supply mechanisms. Western blot analysis was performed to assess the protein levels of Tudor-SN, SM22 and CyclinD1, which serve as indicators of cell contraction and proliferation. As depicted here, Tudor-SN exhibited relatively high expression during this progression (Fig. 1E). Moreover, an immunofluorescence assay using α-SMA as a biomarker of VSMCs showed substantially elevated expression of Tudor-SN within the neointimal area 14 days postsurgery (Fig. 1F). The mean fluorescence intensity and the ratio of the Tudor-SN-to-α-SMA-positive area were separately quantified for analysis (Fig. 1F). In addition, the expression of Tudor-SN was also increased within atherosclerotic plaques of Apoe^−/−^ mice fed with a high-fat diet (Supplemental Fig. 2A), indicating the potential involvement of Tudor-SN in the progression of atherosclerosis.

**Fig. 2:**
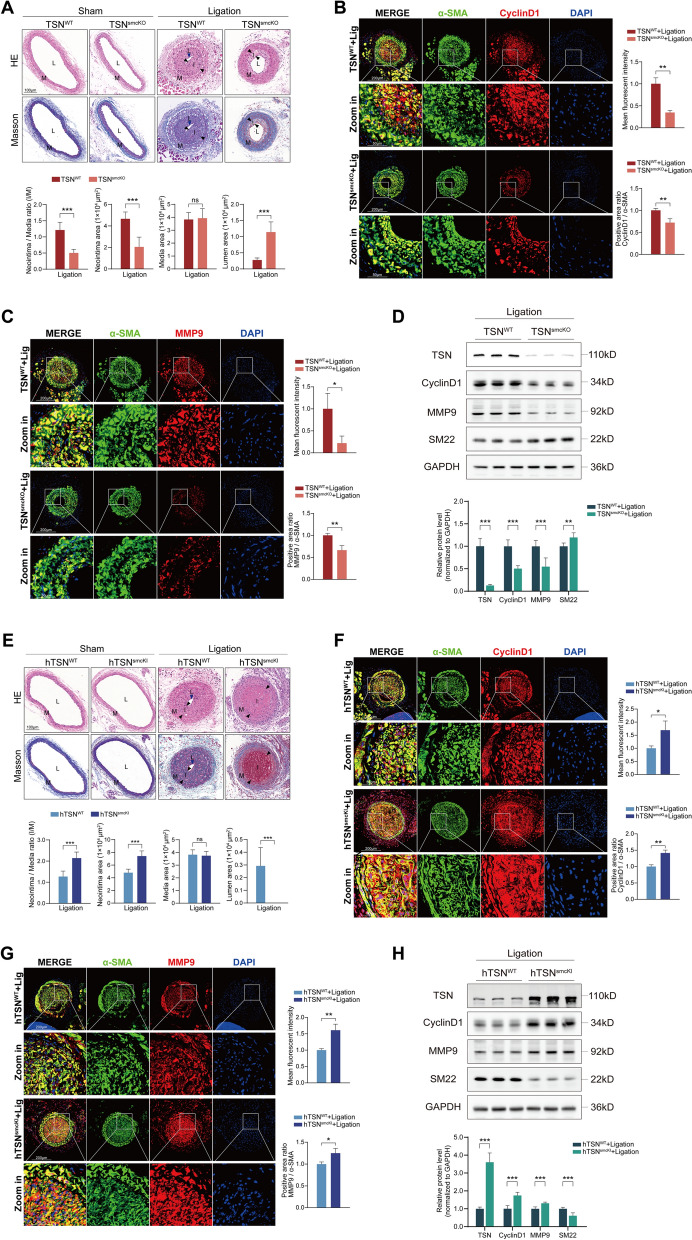
Tudor-SN regulates vascular intimal hyperplasia. **A** H&E and Masson staining of sham-operated and ligated artery sections from TSN^WT^ and TSN^smcKO^ mice 14 days postsurgery (n = 6). L: lumen, indicated by blue arrowheads in the third column; M: media; I: neointima, highlighted by black arrowheads. Scale bar: 100 μm. The statistical analyses of the I/M ratio, neointimal area, media area, and lumen area of the ligated arteries are shown below. **B**, **C** Double immunofluorescence staining of α-SMA (green), CyclinD1 (**B**, red) or MMP9 (**C**, red) in ligated artery sections from TSN^WT^ and TSN^smcKO^ mice 14 days postsurgery (n = 3). Nuclei were stained with DAPI (blue). Scale bar: 200 μm and 50 μm (zoom in). Statistical analyses of CyclinD1 (**B**) or MMP9 (**C**) mean fluorescence intensity and percentage of cells positive for CyclinD1/α-SMA (**B**) or MMP9/α-SMA (**C**) are shown on the right (both were normalized to those in the TSN^WT^ group). **D** Western blot analysis of Tudor-SN, CyclinD1, MMP9, and SM22 in ligated arteries from TSN^WT^ and TSN^smcKO^ mice (n = 6). Statistical analyses of the relative expression levels are shown below. **E** H&E and Masson staining of sham-operated and ligated artery sections from hTSN^WT^ and hTSN^smcKI^ mice 14 days postsurgery (n = 6). L: lumen, indicated by blue arrowheads in the third column; M: media; I: neointima, highlighted by black arrowheads. Scale bar: 100 μm. The statistical analyses of the I/M ratio, neointimal area, media area, and lumen area of the ligated arteries are shown below. **F**, **G** Double immunofluorescence staining of α-SMA (green), CyclinD1 (**F**, red) or MMP9 (**G**, red) in ligated artery sections from hTSN^WT^ and hTSN^smcKI^ mice 14 days postsurgery (n = 3). Nuclei were stained with DAPI (blue). Scale bar: 200 μm and 50 μm (zoom in). Statistical analyses of CyclinD1 (**F**) or MMP9 (**G**) mean fluorescence intensity and percentage of cells positive for CyclinD1/α-SMA (**F**) or MMP9/α-SMA (**G**) are shown on the right (both were normalized to those in the hTSN^WT^ group). **H** Western blot analysis of Tudor-SN, CyclinD1, MMP9, and SM22 in ligated arteries from hTSN^WT^ and hTSN^smcKI^ mice (n = 6). Statistical analyses of the relative expression levels are shown below. Throughout, the results are presented as the mean ± SD; statistical analyses were conducted using Student’s t tests; *p < 0.05, **p < 0.01, ***p < 0.001; *ns* nonsignificant

### Tudor-SN mediates pathological vascular remodeling in mice

As demonstrated by immunofluorescence images, Tudor-SN was predominantly upregulated in VSMCs derived from the original media. Although we cannot exclude the possibility that Tudor-SN functions in endothelium cells or macrophages during vascular intimal hyperplasia, our study prioritized exploring its role in VSMCs. Tudor-SN^fl/fl^; SMMHC-Cre male mice were generated for further experiments, while male littermates with the Tudor-SN^WT/WT^; SMMHC-Cre genotype were used as the control group for validation (Supplemental Fig. 1A); for ease of reference, these mice are referred to as TSN^smcKO^ mice and TSN^WT^ mice, respectively, in the following paragraphs. The level of Tudor-SN in vessels with the adventitia removed were then verified (Supplemental Fig. 1B). Subsequently, CAL was administered to both groups to evaluate the role of Tudor-SN in intimal hyperplasia (Supplemental Fig. 1C). Histological analysis via H&E and Masson staining revealed that genetic ablation of Tudor-SN markedly attenuated neointima formation and collagen deposition compared to that in Tudor-SN^WT^ mice on Day 14 postsurgery (Fig. 2A). Immunofluorescence further revealed a significant reduction in the expression of CyclinD1 and MMP9, key proteins indicative of VSMCs proliferation and migration, within the neointimal area in Tudor-SN^smcKO^ mice (Fig. 2B, C). Western blot experiments using ligated vessels also confirmed downregulation of CyclinD1 and MMP9, accompanied by an increase in SM22 expression (Fig. 2D). To gain further insights into the function of Tudor-SN, hTudor-SN. Stop^fl/−^; SMMHC-Cre male mice were generated for further tests, while male littermates with the hTudor-SN^WT/WT^; SMMHC-Cre genotype were used as the control group for validation (Supplemental Fig. 1E); for ease of reference, these mice are referred to as hTSN^smcKI^ mice and hTSN^WT^ mice, respectively, in the following paragraphs. Western blot analysis demonstrated successful overexpression of Tudor-SN in VSMCs (Supplemental Fig. 1F). H&E staining revealed that Tudor-SN overexpression exacerbated vascular intimal hyperplasia in hTSN^smcKI^ mice, leading to complete vessel occlusion and increased collagen deposition (Fig. 2E). Immunofluorescence experiments were also conducted to assess the expression levels of CyclinD1 and MMP9 in both groups. Consistent with our predictions, the neointimal area of hTSN^smcKI^ mice exhibited significantly greater expression of both proteins than did that of hTSN^WT^ mice (Fig. 2F, G). The ligated vessels were then subjected to western blot analysis, which revealed an upregulation of CyclinD1 and MMP9 upon Tudor-SN overexpression. Conversely, SM22 exhibited an opposite alteration (Fig. 2H). Of note, deletion and overexpression of Tudor-SN also modestly altered the expression levels of CyclinD1 and MMP9 in the vessel wall of sham-operated arteries (Supplemental Fig. 3A-D). These findings suggest a prominent regulatory role of Tudor-SN in vascular intimal hyperplasia following vascular injury and its potential impact on the proliferation and migration of VSMCs. Furthermore, Tudor-SN-deficient mice (*Apoe*^*−/−*^) were generated to investigate its role in atherosclerosis progression. An en-face Oil Red O staining of the entire aorta showed significantly reduced plaque area in TSN^smcKO^ mice compared to the control group (Supplemental Fig. 2B). Correspondingly, the percentage of plaque area in the aortic root also decreased upon Tudor-SN knockout (Supplemental Fig. 2C). Subsequently, an evaluation of plaque stability was conducted and TSN^smcKO^ mice exhibited decreased necrotic core with increased collagen content (Supplemental Fig. 2D). Collectively, these results indicate a potential role of Tudor-SN in regulating atherosclerosis development and plaque stabilization.

### Tudor-SN regulates PDGF-BB-induced phenotypic switching in mouse smooth muscle cells

**Fig. 3:**
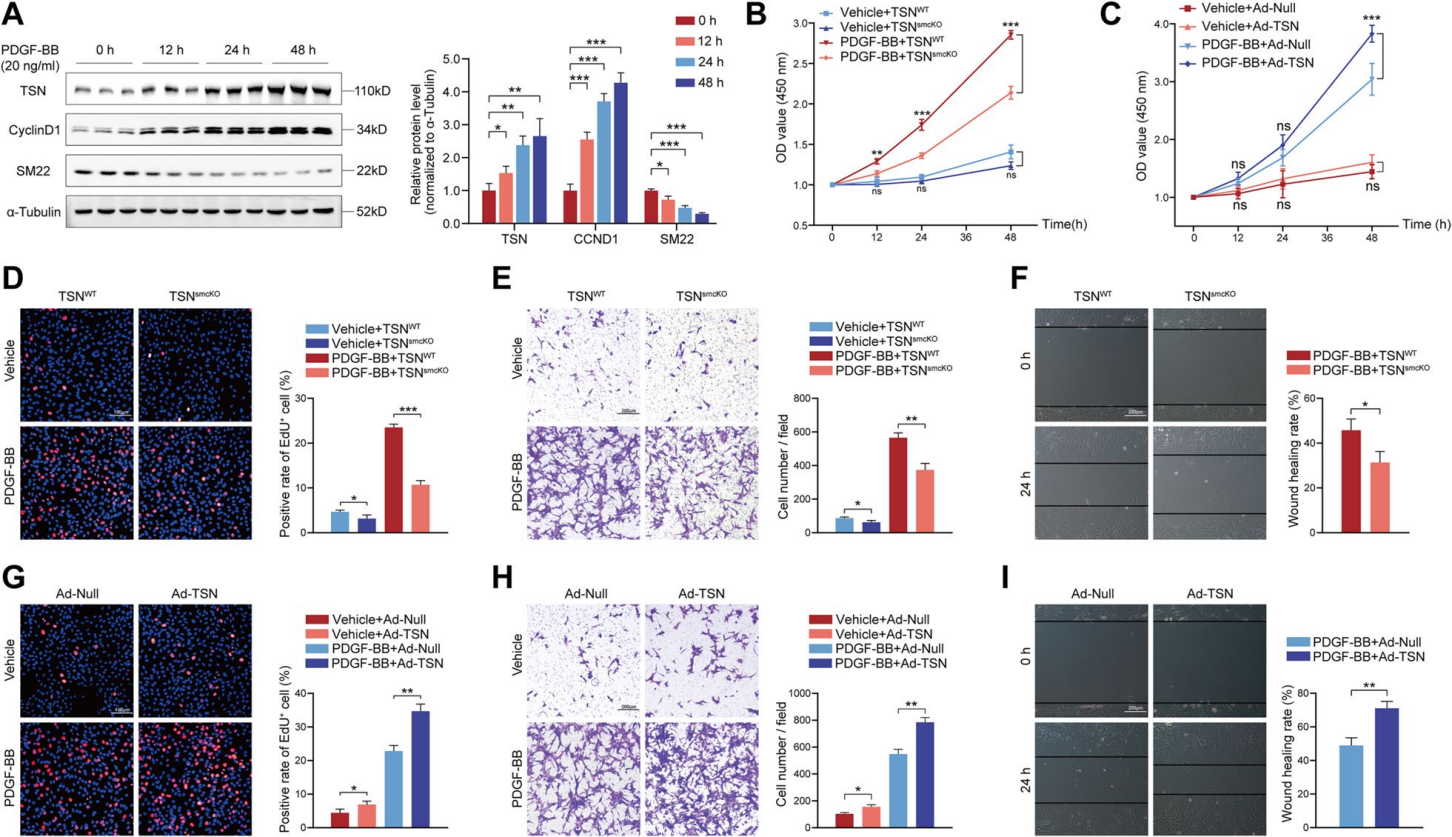
Tudor-SN mediates the phenotypic switching of MASMCs. **A** Western blot analysis and statistical analysis of Tudor-SN, CyclinD1, and SM22 expression in MASMCs at different time intervals after PDGF-BB induction. **B**, **D**, **E**, **F** Primary MASMCs from TSN^WT^ and TSN^smcKO^ mice were cultured and treated with vehicle or PDGF-BB (20 ng/ml). Cell proliferation was examined using a CCK-8 assay (**B**) and EdU staining (**D**, scale bar: 100 μm). The percentage of EdU^+^ cells was analyzed in the statistical chart. Cell migration was tested using transwell (**E**) and wound healing (**F**) assays. The cell count per field and wound healing rate were analyzed. Scale bar: 200 μm. **C**, **G**, **H**, **I** Primary MASMCs from wild-type mice were cultured and treated with Ad-Null/ Ad-TSN before exposed to vehicle or PDGF-BB (20 ng/ml). Cell proliferation was examined using a CCK-8 assay (**C**) and EdU staining (**G**, scale bar: 100 μm). The percentage of EdU^+^ cells was evaluated in the statistical chart. Cell migration was tested using transwell (**H**) and wound healing (**I**) assays. The cell count per field and wound healing rate were analyzed. Scale bar: 200 μm. The results are representative of at least three independent experiments and are shown as the mean ± SD; statistical analyses were conducted using Student’s t tests in data F & I, one- or two-way ANOVA with a post hoc test of Tukey’s analysis in data A/D/E/G/H and B/C, respectively; *p < 0.05, **p < 0.01, ***p < 0.001; *ns* nonsignificant

Given that VSMCs in the tunica media play a central role in neointima formation, we speculated that Tudor-SN may be involved in the regulation of VSMCs phenotypic switching. Platelet-derived growth factor-BB (PDGF-BB) has been widely acknowledged for its ability to induce the transition of VSMCs toward a synthetic phenotype [18]. Western blot analysis revealed a remarkable increase in Tudor-SN expression upon PDGF-BB stimulation (Fig. 3A), suggesting the plausible involvement of Tudor-SN in this process. Initially, tamoxifen was used to induce Tudor-SN knockout in primary mouse aortic smooth muscle cells (MASMCs). Western blot experiments were also conducted on TSN^smcKO^ and TSN^WT^ cells, and it was noted that some residual stripes were observed in the TSN^smcKO^ cells, which could be attributed to variations in tamoxifen concentration [19] or potential technical limitations resulting in minimal contamination by vascular endothelial cells (Supplemental Fig. 1D). CCK-8 and EdU experiments were carried out to detect the proliferative capacity of the cells before and after PDGF-BB (20 ng/ml) stimulation. Consistent with the in vivo results, Tudor-SN knockdown effectively suppressed MASMCs proliferation compared to that in the control group (Fig. 3B, D). To evaluate migratory capacity, transwell and wound healing assays were subsequently performed. The results also demonstrated that Tudor-SN knockdown had a critical inhibitory effect on MASMCs migration (Fig. 3E, F). Additionally, an adenovirus overexpressing Tudor-SN was constructed and tested on MASMCs isolated from wild-type mice through Western blot analysis (Supplemental Fig. 1H). Similarly, CCK-8, EdU, transwell and wound healing assays revealed the opposite effects when compared to Tudor-SN knockout groups, wherein the overexpression of Tudor-SN significantly enhanced the proliferation and migration of MASMCs (Fig. 3C, G, H, I). The collagen gel contraction assay was also performed and evidenced that Tudor-SN-deficient cells possessed stronger contractile abilities (Supplemental Fig. 4A). Notably, in the absence of PDGF-BB stimulation, the loss and gain of Tudor-SN resulted in a modest decrease and increase, respectively, in MASMCs proliferation and migration abilities. During cell culture and passaging, MASMCs can convert from a quiescent state to a synthetic state without additional stimuli. It is also plausible that the effect of Tudor-SN is dramatically potentiated by strong stimuli, such as growth factors.

To further explore the underlying mechanism of Tudor-SN upregulation following PDGF-BB stimulation, the mRNA level was examined and found to be increased after PDGF-BB stimulation (data not shown). By interrogating databases including AnimalTFDB, PROMO, hTF target, JASPAR and CHEA, putative upstream transcription factors of Tudor-SN were identified and visualized in a Venn diagram (Supplemental Fig. 4B). Several potential factors were selected for further tests. Interestingly, the results of luciferase reporter assays showed that CREB1 could dramatically increase the Tudor-SN luciferase activity (Supplemental Fig. 4C). More importantly, knockdown of CREB1 reversed the PDGF-BB-induced upregulation in mRNA level of Tudor-SN (data not shown), suggesting it may be the core transcriptional factor in mediating the Tudor-SN transcription under PDGF-BB treatment.

### Tudor-SN aggravates PDGF-BB-induced effects via the MAPK and PI3K-AKT signaling pathways

**Fig. 4:**
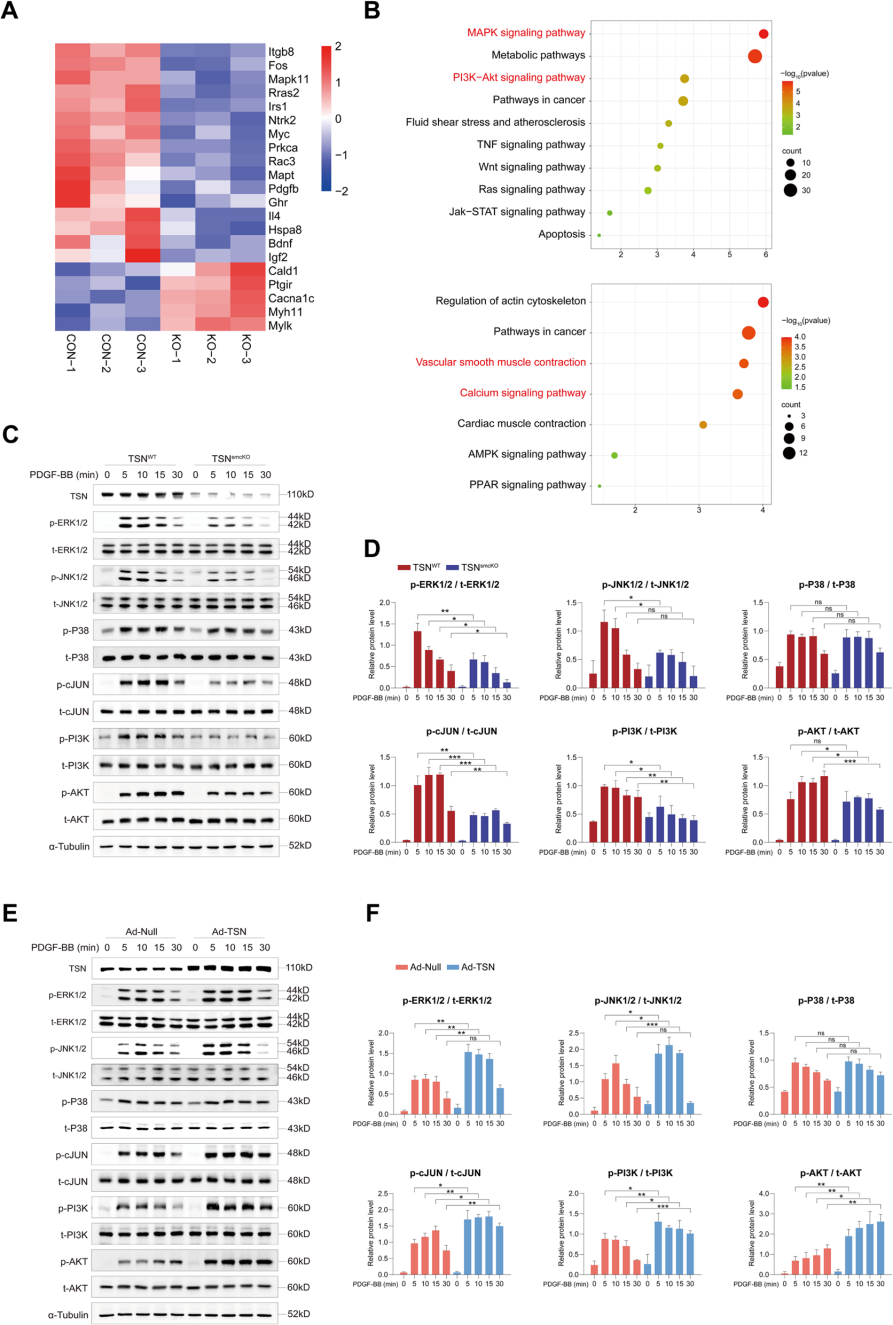
Tudor-SN affects the MAPK and PI3K-AKT signaling pathways in MASMCs **A** Heatmap of genes of interest whose expressions were differentially expressed in aortas from TSN^WT^ (CON) and TSN^smcKO^ (KO) mice identified through transcriptome sequencing (n = 3). **B** KEGG enrichment analysis of downregulated genes (up) and upregulated genes (bottom) in TSN^smcKO^ mice. The terms of interest are marked in red. **C**, **E** Western blot analysis of several MAPK and PI3K-AKT signaling pathway components (n = 3). Primary MASMCs were isolated from TSN^WT^ and TSN^smcKO^ mice (**C**) or wild-type mice treated with adenoviruses (**E**). Cell proteins were extracted at different time intervals after PDGF-BB stimulation. **D**, **F** The activation levels of ERK1/2, JNK1/2, P38, c-JUN, PI3K, and AKT were assessed by calculating the ratio of phosphorylated protein to total protein. Throughout, the results are presented as the mean ± SD; statistical analyses were conducted using one-way ANOVA with a post hoc test of Tukey’s analysis; *p < 0.05, **p < 0.01, ***p < 0.001; *ns* nonsignificant

To elucidate the underlying mechanism of Tudor-SN, RNA sequencing of aortas (with the adventitia removed) isolated from both TSN^WT^ and TSN^smcKO^ mice was subsequently performed. A total of 32,134 DEGs were identified, among which 376 genes exhibited pronounced upregulation and 394 genes showed significant downregulation in the TSN^smcKO^ group (Supplemental Fig. 5A). An overall clustering of the differentially expressed genes was obtained (Supplemental Fig. 5B). Notably, differences in the expression levels of certain genes were closely associated with classical signaling pathways implicated in vascular intimal hyperplasia (Fig. 4A). Kyoto Encyclopedia of Genes and Genomes (KEGG) analysis revealed significant enrichment of downregulated genes within the MAPK and PI3K-AKT signaling pathways in the TSN^smcKO^ group (Fig. 4B up). Conversely, the upregulated genes were predominantly enriched in signaling pathways associated with vascular smooth muscle contraction (Fig. 4B bottom). Gene Ontology (GO) analysis indicated that the downregulated genes were involved in various biological processes, including VSMCs proliferation and migration, extracellular matrix formation, and positive regulation of the MAPK and PI3K signaling pathways (Supplemental Fig. 5C). These results strongly supported the regulatory role of Tudor-SN during the phenotypic switching of VSMCs. In contrast, the upregulated genes were primarily related to vasoconstriction, smooth muscle contraction, and negative regulation of cell proliferation and migration (Supplemental Fig. 5D), which was consistent with our previous findings. Considering the essential role of the MAPK and PI3K-AKT signaling pathways in pathological vascular remodeling and VSMCs phenotypic switching [6, 20], these findings prompt speculation on whether Tudor-SN participates in regulating the MAPK and PI3K-AKT signaling pathways under pathological conditions. Therefore, cellular proteins from the TSN^WT^ and TSN^smcKO^ groups were extracted at 0, 5, 10, 15, and 30 min after PDGF-BB stimulation for subsequent Western blot experiments. The results demonstrated rapid activation of the ERK1/2, JNK1/2, P38, c-JUN, and PI3K-AKT pathways during the early stage. Loss of Tudor-SN resulted in diminished levels of phosphorylated ERK1/2, JNK1/2, c-JUN, PI3K and AKT relative to the total protein content (Fig. 4C, D). However, the activation level of the P38 pathway remained unaffected. In contrast, when Tudor-SN was overexpressed through adenovirus delivery in wild-type MASMCs and subjected to Western blot analysis under identical conditions, increased phosphorylation levels of the aforementioned pathways were detected, while the P38 pathway was not altered (Fig. 4E, F). Taken together, our findings highlighted the crucial role of Tudor-SN in orchestrating MAPK and PI3K-AKT activation following PDGF-BB stimulation.

### Tudor-SN accelerates the degradation of PTEN through interaction

**Fig. 5:**
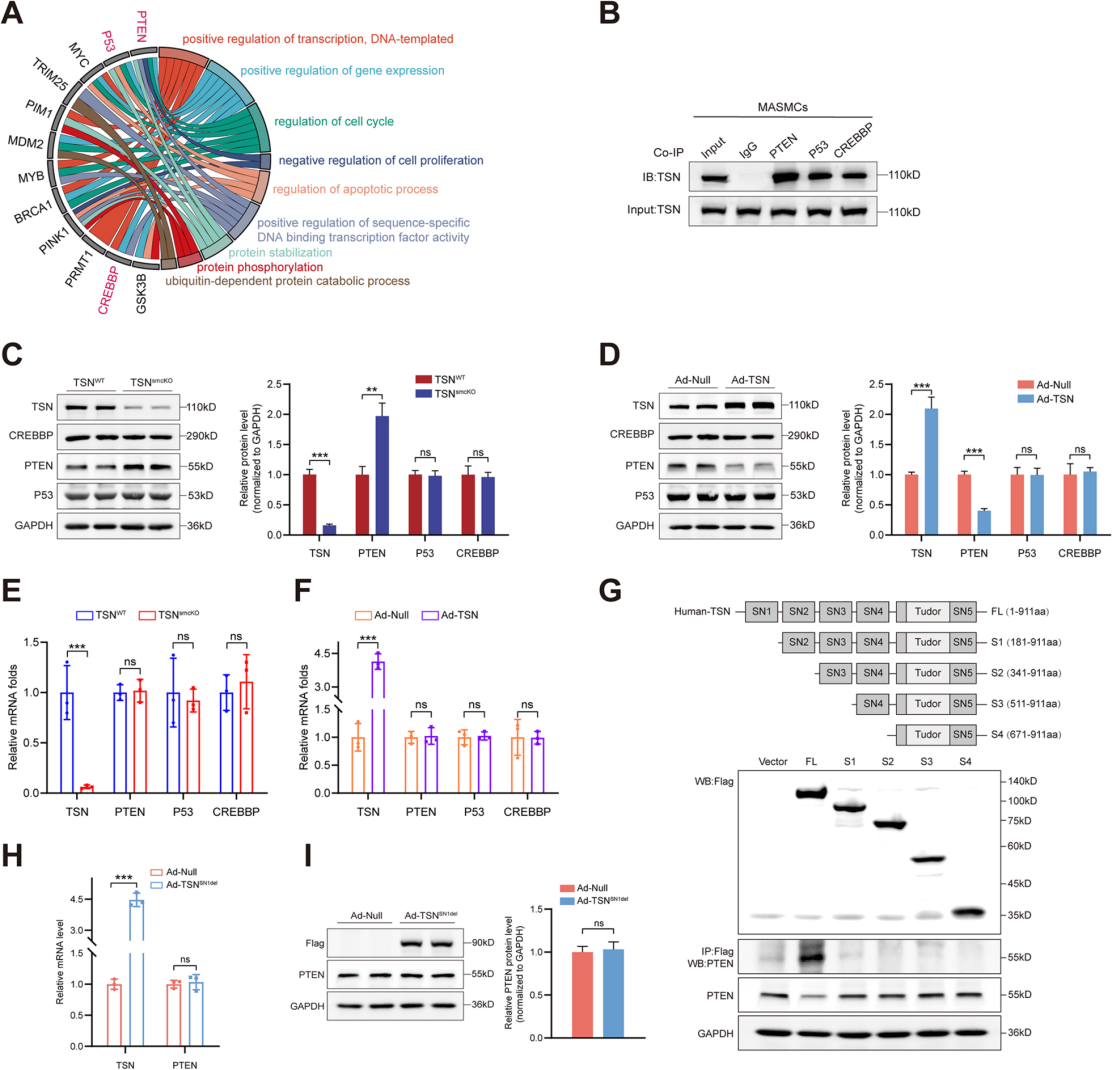
Tudor-SN interacts with PTEN through the SN1 domain and facilitates its degradation. **A** The GO chordal graph demonstrated that several proteins are known to interact with Tudor-SN. The data were collected from both human and mouse spicies using the BioGRID database. The proteins of interest are marked in red. **B** The interactions between Tudor-SN and PTEN, P53, and CREBBP were determined by coimmunoprecipitation assays in MASMCs (n = 3). **C**, **D** Western blot analyses of PTEN, P53, and CREBBP in primary TSN^WT^ and TSN^smcKO^ MASMCs (**C**, n = 3) and in wild-type MASMCs treated with Ad-Null or Ad-TSN (**D**, n = 3). **E**, **F** The mRNA levels of PTEN, P53, and CREBBP were determined by RT‒PCR in primary TSN^WT^ and TSN^smcKO^ MASMCs (**E**, n = 3) and wild-type MASMCs treated with Ad-Null or Ad-TSN (**F**, n = 3). **G** HEK293T cells were transfected with plasmids expressing different domains of Tudor-SN and subjected to coimmunoprecipitation (n = 3). **H**, **I** The mRNA and protein levels of PTEN were assessed after treatment with a mutant adenovirus (Ad-TSN^SN1del^) (n = 3). Throughout, the results are presented as the mean ± SD; statistical analyses were conducted using Student’s t tests; *p < 0.05, **p < 0.01, ***p < 0.001; *ns* nonsignificant

To better elucidate how Tudor-SN functions as a cellular fate regulator, GO enrichment analysis was performed using the BioGRID database to identify proteins known to interact with Tudor-SN. Data from both human and mouse species were collected, and the proteins of interest were screened out as potential targets of Tudor-SN and depicted in a chordal graph (Fig. 5A). Among them, PTEN [21–24], P53 [25, 26], MYC [27, 28], PIM1 [29], MYB [30], PINK1 [31, 32], PRMT1 [33], CREBBP [34, 35], and GSK3B [36] have been reported to be involved in regulating VSMCs phenotypic switching. Specifically, PTEN [37, 38], P53 [39–41], and CREBBP [42, 43] are closely associated with the MAPK and PI3K-AKT signaling pathways. Endogenous coimmunoprecipitation experiments were conducted in wild-type MASMCs, and all three proteins were found to interact with Tudor-SN (Fig. 5B). Therefore, whether Tudor-SN was involved in altering PTEN, P53, and CREBBP at the posttranslational level were further investigated. A significant increase in the PTEN protein level upon loss of Tudor-SN was observed, while P53 and CREBBP remained unchanged (Fig. 5C). In contrast, gain of Tudor-SN in wild-type MASMCs led to a notable decrease in the PTEN protein level but did not affect the protein levels of P53 or CREBBP (Fig. 5D). Given the well-established role of Tudor-SN at the transcriptional level, subsequent real-time polymerase chain reactions (RT–PCRs) were carried out. Interestingly, the mRNA levels of PTEN, P53, and CREBBP did not change upon either loss or gain of Tudor-SN (Fig. 5E, F), suggesting that the impact of Tudor-SN on PTEN expression was likely achieved through interaction at the protein level. As a widely documented tumor suppressor protein, PTEN has been extensively characterized for its multifunctional roles in VSMCs. Ata et al. reported that PTEN enhances the Ca^2+^ concentration in VSMCs by activating the PKC pathway, thereby promoting vasoconstriction [21]. Horita et al. established that PTEN interacts with serum response factor (SRF) and facilitates the binding of SRF to specific downstream genes to maintain the contractile phenotype of VSMCs [23]. Furgeson et al. demonstrated that loss of PTEN in VSMCs aggravated intimal hyperplasia through enhancing phosphorylation of the downstream PI3K-AKT signaling pathway [22]. Moreover, PTEN is involved in regulating the proliferation and migration of VSMCs during atherosclerosis progression [24], and its functions in modulating the phosphorylation of ERK/JNK pathways have been confirmed across various disease models [37, 44]. Overall, we hypothesized that PTEN plays a vital role in Tudor-SN-mediated regulation of VSMCs.

For further exploration, our objective was to validate the specific interaction domain between Tudor-SN and PTEN. Different truncated forms of human Tudor-SN were generated on an overexpression plasmid tagged with 3 × Flag: full length (FL, 1–911 aa), S1 (181–911 aa), S2 (341–911 aa), S3 (511–911 aa), and S4 (671–911 aa). HEK293T cells were subsequently transfected with these plasmids and subjected to coimmunoprecipitation experiments. Notably, only the FL-Tudor-SN group showed a positive outcome, pinpointing the PTEN-binding site within the SN1 domain of Tudor-SN (Fig. 5G). In agreement with these findings, the application of a mutant Tudor-SN adenovirus with the SN1 domain deleted (TSN^SN1del^) did not result in significant alterations in either the mRNA or protein levels of PTEN (Fig. 5H, I).

**Fig. 6:**
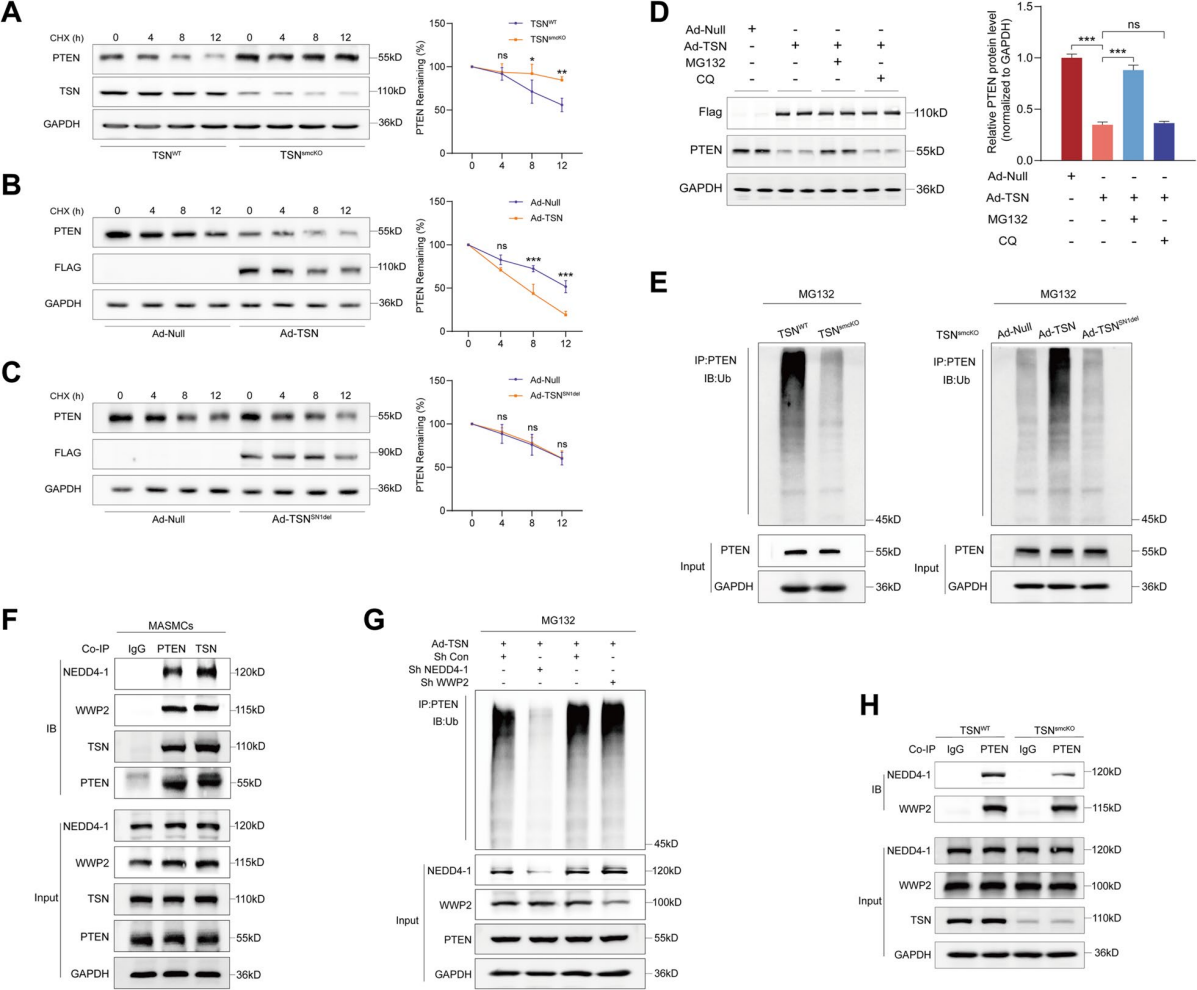
Tudor-SN promotes PTEN polyubiquitination via NEDD4-1. **A**, **B**, **C** Western blot analysis of PTEN degradation in cells treated with cycloheximide (CHX) (n = 3). Primary MASMCs were isolated from TSN^WT^ or TSN^smcKO^ mice (**A**) and wild-type mice treated with Ad-Null, Ad-TSN or Ad-TSN^SN1del^ (**B**, **C**). The protein half-life curves are shown on the right. (**D**) The protein level of PTEN was detected in MASMCs treated with MG132 or CQ (n = 3). **E** Results from the PTEN polyubiquitination assays after silencing (left) or overexpressing different forms of Tudor-SN (right). **F** The interactions between Tudor-SN, PTEN, NEDD4-1, and WWP2 in MASMCs were detected using coimmunoprecipitation assays (n = 3). **G** The effects of NEDD4-1 and WWP2 silencing on Tudor-SN-mediated PTEN polyubiquitination in MASMCs (n = 3). **H** Effects of Tudor-SN deletion on the interactions between PTEN, NEDD4-1, and WWP2 (n = 3). Throughout, the results are presented as the mean ± SD; statistical analyses were conducted using one- or two-way ANOVA with a post hoc test of Tukey’s analysis in data D and A/B/C, respectively; *p＜0.05, **p < 0.01, ***p < 0.001; *ns* nonsignificant

Next, differently treated MASMCs were exposed to cycloheximide (CHX) to inhibit translation, after which the degradation rate of the synthesized proteins was observed. Cellular proteins were extracted at four time points (0 h, 4 h, 8 h, and 12 h) for subsequent analysis. Here, a dramatic decrease in the PTEN degradation rate was observed in TSN^smcKO^ cells compared to that in the control group (Fig. 6A). Conversely, overexpression of Tudor-SN clearly accelerated the PTEN degradation rate (Fig. 6B). However, no substantial change in the PTEN degradation rate was detected in cells overexpressing TSN^SN1del^ (Fig. 6C). This evidence supported the notion that Tudor-SN interacts with PTEN and facilitates its protein degradation through the SN1 domain.

Given that intracellular protein degradation occurs primarily through either the autophagy-lysosomal or ubiquitin‒proteasome pathway in eukaryotic cells [45, 46], our subsequent investigation aimed to clarify the specific mechanism by which Tudor-SN impacts PTEN degradation. By employing chloroquine (CQ), a lysosomal inhibitor, or MG132, a proteasome inhibitor, we demonstrated that MG132 significantly impeded PTEN degradation mediated by Tudor-SN, while CQ did not (Fig. 6D), suggesting that Tudor-SN might serve as a mediator of the ubiquitin‒proteasome pathway.

However, to date, few studies have elucidated the intricate relationship between Tudor-SN and ubiquitination. Zhan et al. reported that Tudor-SN participated in SMURF1-mediated ubiquitination of FOXA2, thus promoting enhanced epithelial–mesenchymal transition in cervical cancer [47]. Additionally, Rajasekaran et al. revealed that Tudor-SN interacts with MGLL, facilitating its ubiquitination and subsequent degradation in hepatocellular carcinoma (HCC) [48]. Based on the results of existing studies, we postulated that Tudor-SN may modulate PTEN protein levels by influencing its ubiquitination process.

### Tudor-SN facilitates the polyubiquitination of PTEN via NEDD4-1

To further decipher the role of Tudor-SN in PTEN ubiquitination, both TSN^WT^ and TSN^smcKO^ cells were treated with MG132 and assessed for endogenous ubiquitination of PTEN. Immunoprecipitation assays revealed a significant decrease in the polyubiquitination of PTEN in TSN^smcKO^ cells compared to that in TSN^WT^ cells (Fig. 6E, left), while overexpression of Tudor-SN, but not TSN^SN1del^, markedly restored PTEN polyubiquitination in TSN^smcKO^ cells (Fig. 6E, right). Since PTEN is a strictly regulated molecule in eukaryotic cells and the specific role of Tudor-SN in protein ubiquitination has not been determined, we postulated the involvement of another key molecule between Tudor-SN and PTEN. To date, NEDD4-1 and WWP2 are two well-characterized E3 ubiquitin ligases that trigger the degradation of PTEN [49–52]. We subsequently hypothesized that Tudor-SN may facilitate the interaction between these E3 ubiquitin ligases and PTEN to exert its regulatory influence on PTEN ubiquitination. To verify this hypothesis, an endogenous coimmunoprecipitation assay was initially performed with wild-type MASMCs, confirming pairwise interactions between Tudor-SN and PTEN with both NEDD4-1 and WWP2 (Fig. 6F). Adenoviruses were subsequently generated to knock down NEDD4-1 and WWP2, and immunoprecipitation experiments showed that the increase in Tudor-SN-mediated PTEN polyubiquitination was significantly abolished upon the loss of NEDD4-1, while WWP2 knockdown had no impact (Fig. 6G). These findings suggested the potential dependence of Tudor-SN-induced enhancement of PTEN degradation on NEDD4-1. Thus, we further proposed that Tudor-SN may play a role in mediating the interactions between PTEN and these E3 ubiquitin ligases. Coimmunoprecipitation experiments conducted on TSN^WT^ and TSN^smcKO^ cells provided supportive evidence that the combination of NEDD4-1 and PTEN was significantly decreased in TSN^smcKO^ cells, while the combination of WWP2 and PTEN was not changed (Fig. 6H). Overall, we could infer that Tudor-SN was required for facilitating the interaction between NEDD4-1 and PTEN.

### Silencing PTEN abrogates the effects of Tudor-SN deletion both in vivo and in vitro

**Fig. 7:**
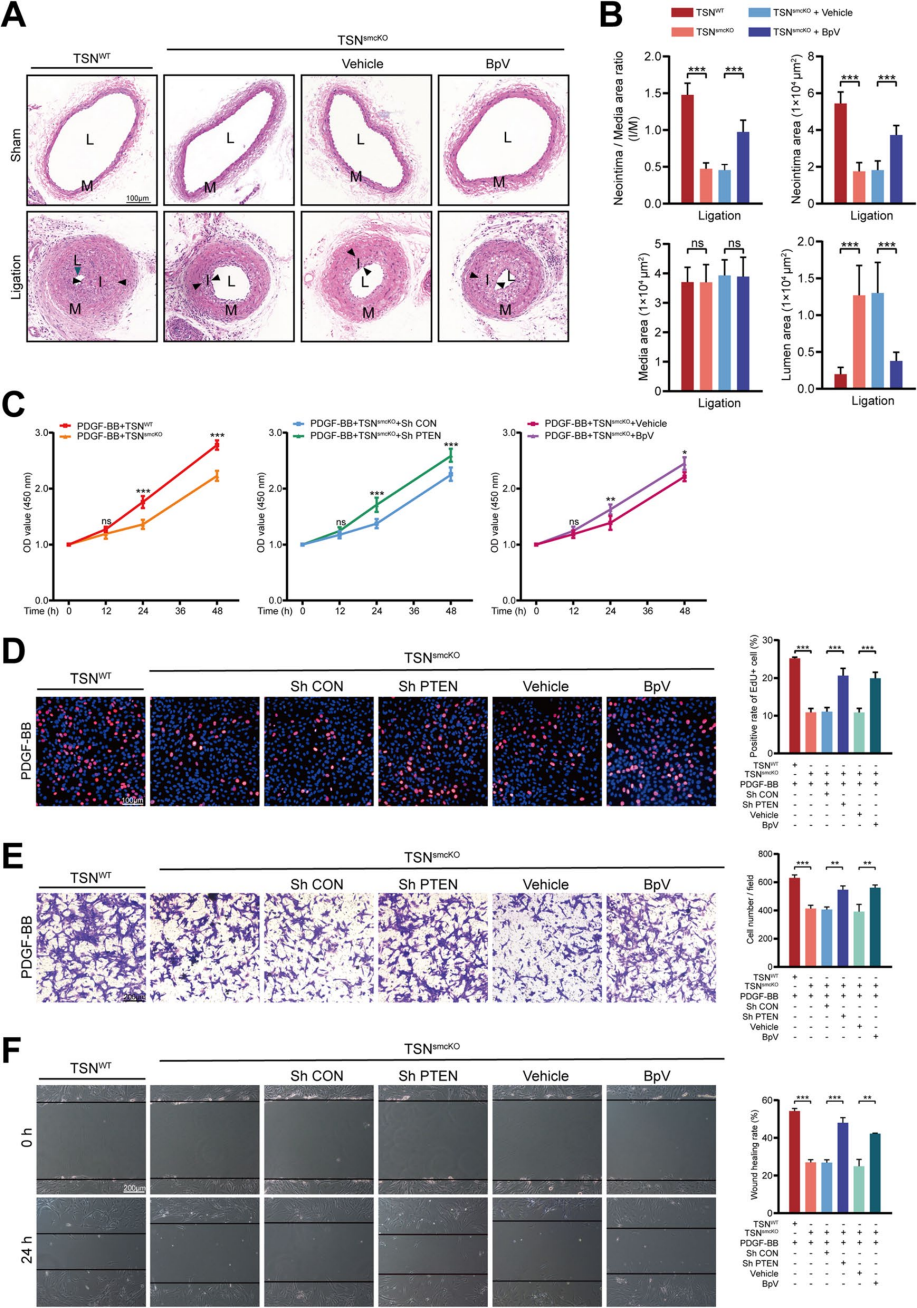
PTEN interference abrogates the effects of Tudor-SN deletion. **A** H＆E staining of sham-operated and ligated artery sections from mice treated with vehicle or BpV 14 days postsurgery (n = 6). L: lumen, indicated by blue arrowheads in the first column; M: media; I: neointima, highlighted by black arrowheads. Scale bar: 100 μm. **B** Statistical analyses of the I/M ratio, neointimal area, media area, and lumen area of the ligated arteries. **C**, **D** The effects of PTEN knockdown and inhibition on cell proliferation in Tudor-SN-deleted MASMCs were examined using a CCK-8 assay (**C**) and EdU staining (**D**, scale bar: 100 μm). The percentage of EdU^+^ cells was analyzed in the statistical chart. **E**, **F** The effects of PTEN knockdown and inhibition on cell migration were tested using transwell (**E**) and wound healing (**F**) assays. The cell count per field and wound healing rate were analyzed. Scale bar: 200 μm. Throughout, the results are presented as the mean ± SD; statistical analyses were conducted using one- or two-way ANOVA with a post hoc test of Tukey's analysis in data B/D/E/F and C, respectively; *p＜0.05, **p＜0.01, ***p＜0.001; *ns* nonsignificant

**Fig. 8:**
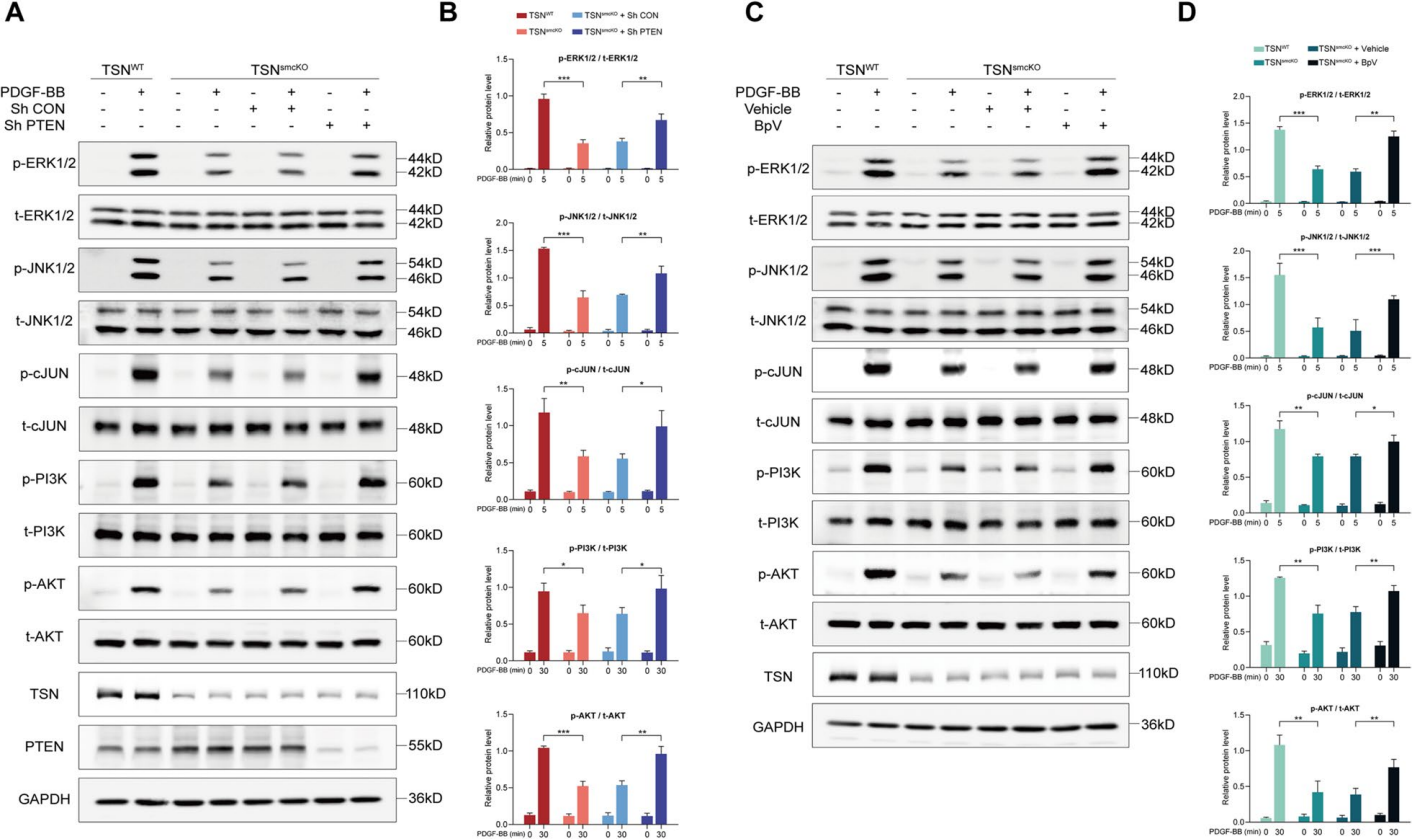
PTEN interference reverses the effects of Tudor-SN on MAPK and PI3K-AKT signaling pathways. **A**, **B**, **C**, **D** The effects of PTEN knockdown (**A**) and inhibition (**C**) on the activation of the ERK1/2, JNK1/2, P38, c-JUN, PI3K, and AKT signaling pathways in primary TSN^WT^ and TSN^smcKO^ MASMCs (n = 3). The statistical charts are shown on the right, respectively (**B**, **D**). Throughout, the results are presented as the mean ± SD; statistical analyses were conducted using one-way ANOVA with a post hoc test of Tukey’s analysis in data B and D; *p < 0.05, **p < 0.01, ***p < 0.001; *ns* nonsignificant

To further substantiate our inference, we conducted interventions to manipulate the expression and activity of PTEN to determine its potential reversible role. BpV (HOpic), an extensively utilized selective PTEN inhibitor [53, 54], was initially administered via intraperitoneal injection at a dosage of 0.5 mg/kg per day to inhibit PTEN activity in TSN^smcKO^ mice, while the control group received saline solution (vehicle). CAL was performed, and compared with those in the vehicle group, the I/M ratio and neointimal area were significantly greater in the BpV injection group, whereas the lumen area was decreased (Fig. 7A, B). These findings indicated that blocking PTEN activity could partially reverse the inhibitory effect of Tudor-SN deletion on vascular intimal hyperplasia. For in vitro studies, MASMCs isolated from TSN^smcKO^ mice were exposed to BpV (0.5 μM) or an adenovirus for PTEN interference. The results of CCK-8 and EdU assays demonstrated that both the knockdown and inhibition of PTEN partially reversed the inhibitory effects of Tudor-SN loss on cell proliferation (Fig. 7C, D). Consistently, transwell and wound healing assays revealed similar effects on cell migration when PTEN was knocked down or inactivated (Fig. 7E, F). More importantly, both interventions were found to partly reverse the suppressive effects of Tudor-SN deficiency on the aforementioned signaling pathways (ERK1/2, JNK/2, c-JUN, PI3K, and AKT) to some extent (Fig. 8A–D). Together, these results highlighted the dependency of Tudor-SN on PTEN.

**Fig. 9:**
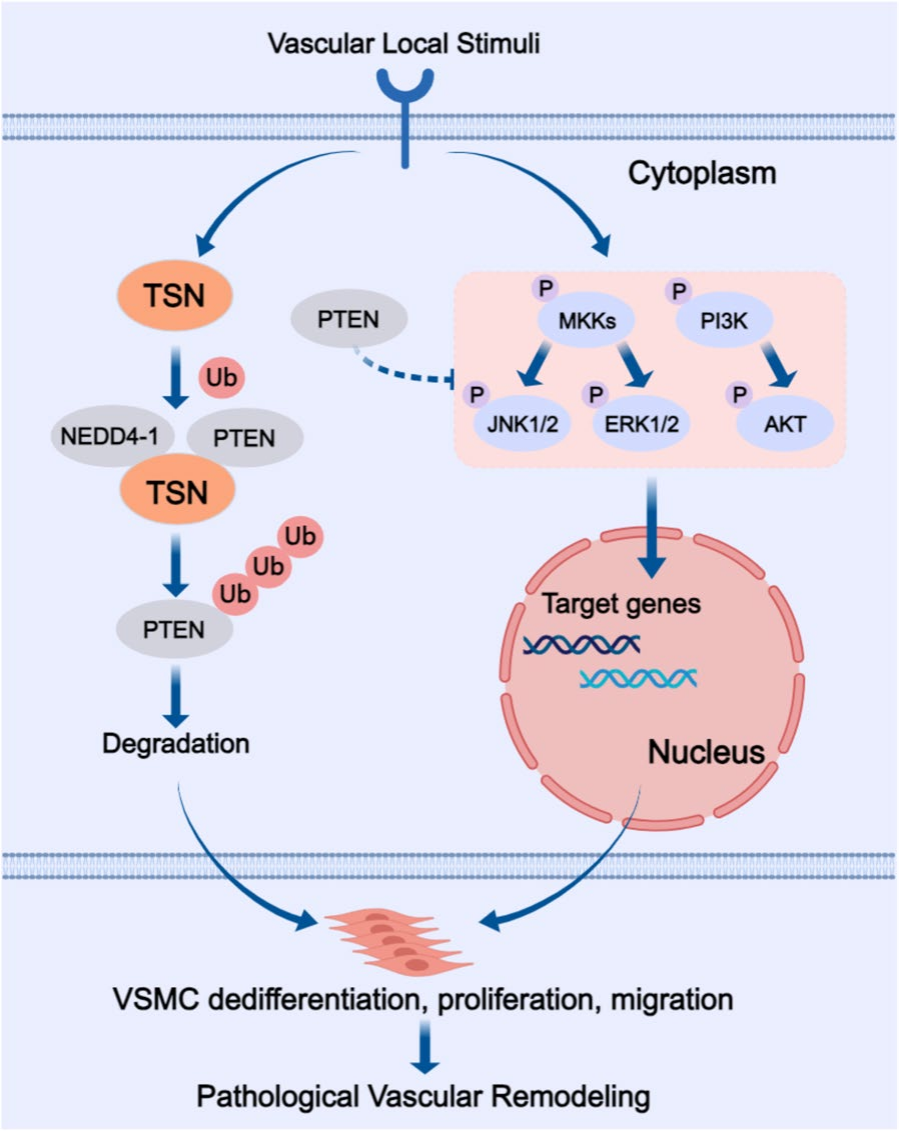
Schematic showing the mechanism by which Tudor-SN mediates vascular remodeling. The expression of Tudor-SN is upregulated in VSMCs following vascular local stimuli, leading to enhanced polyubiquitination and degradation of PTEN via NEDD4-1. The interaction between Tudor-SN and PTEN promotes downstream signaling pathways, resulting in over-activation of MAPK and PI3K-AKT pathways that ultimately exacerbates VSMCs’ dedifferentiation, proliferation, migration and pathological vascular remodeling

In summary, our study has unveiled a significant role of Tudor-SN as a regulator of cellular fate during pathological vascular remodeling through influencing the degradation of PTEN. When exposed to local stimuli, Tudor-SN is upregulated in VSMCs and serves as an intermediary between PTEN and NEDD4-1. By facilitating the polyubiquitination of PTEN via NEDD4-1, Tudor-SN accelerates PTEN degradation, thereby liberating downstream signaling pathways and promoting the phenotypic switching of VSMCs, eventually leading to pathological remodeling of blood vessels (Fig. 9). Our findings propose that targeting Tudor-SN could be a promising therapeutic strategy for further investigation aimed at preventing vascular diseases.

## Discussion

Percutaneous coronary intervention (PCI) is currently the foremost approach for treating coronary atherosclerotic heart disease. However, damage-induced pathological vascular remodeling is primarily responsible for the occurrence of in-stent restenosis (ISR) following PCI [55, 56]. Despite the annual use of millions of drug-eluting stents to reduce the incidence of ISR, it still affects 5%-10% of patients and remains a critical clinical issue that needs resolution [57, 58]. Here, we observed a positive correlation between Tudor-SN expression and the expression of a series of genes associated with VSMCs proliferation and migration in human normal coronary arteries. Further analysis revealed the upregulation of Tudor-SN expression in both human BMS-implanted LIMA segments and carotid atherosclerotic plaques. Based on these new findings, experiments were designed to evaluate the potential role of Tudor-SN in pathological vascular remodeling.

Tudor-SN is known for its high conservation and comprises four consecutive repeats of Staphylococcal nuclease-like domains (referred to as SN1-SN4) at the N-terminus, a Tudor domain at the C-terminus, and a fusion of part of the SN5 domain with the Tudor structure. This multidomain architecture endows Tudor-SN with diverse biological functions. Initially, this molecule was identified as a transcriptional coactivator for Epstein-Barr nuclear antigen 2 (EBNA2) [12] and has been identified as a coactivator of PIM-1 [59], STAT6 [60–62], PPARγ [63], E2F1 [64], and HIF1-α [65] in subsequent research. Moreover, Tudor-SN exerts post-transcriptional effects by attenuating mRNA degradation [66] and facilitating RISC-dependent gene silencing [16]. Recent studies on Tudor-SN have predominantly focused on tumors, with several reports revealing upregulated expression of Tudor-SN in various human tumor cells [13–16]. Notably, this molecule has been positively associated with distinct tumor metastasis events and unfavorable prognosis [13, 16, 67, 68]. For instance, Tudor-SN is believed to impact the clinical progression of HCC [16] and promote angiogenesis through the nuclear factor κB (NF-κB) signaling pathway [69]. However, whether Tudor-SN participates in vascular remodeling is poorly understood.

Pathological phenotypic switching caused by vascular damage induces VSMCs to proliferate and migrate from the initial media into the vessel lumen, releasing a substantial amount of extracellular matrix and leading to the development of aberrant neointimal lesions. Here, we identified a significant increase in Tudor-SN expression in a mouse CAL model. VSMCs lacking Tudor-SN exhibited impaired proliferative and migratory abilities upon vascular injury, resulting in reduced neointimal lesions. Notably, in vitro studies further substantiated the capacity of this molecule to affect VSMCs proliferation and migration under both physiological and pathological circumstances. Additionally, specific gain of Tudor-SN resulted in contrasting effects, as aggravated vascular remodeling was observed.

To further explore the role of Tudor-SN in vascular diseases, we performed RNA sequencing on aortas from TSN^WT^ and TSN^smcKO^ mice. KEGG analysis revealed that Tudor-SN may function as a mediator of the MAPK and PI3K-AKT signaling pathways. Moreover, the upregulated genes upon Tudor-SN deletion were closely associated with vascular smooth muscle contraction. According to further GO analysis, vessels lacking Tudor-SN exhibited decreased expression of genes involved in VSMCs proliferation and migration, extracellular matrix formation, and positive regulation of the MAPK and PI3K signaling pathways. Conversely, genes associated with vasoconstriction, smooth muscle contraction, and negative regulation of cell proliferation and migration showed elevated expression levels. These findings were highly consistent with the observed effects exerted by Tudor-SN, suggesting that Tudor-SN may serve as a potent novel mediator in the progression of vascular diseases.

Mechanistically, the activation of the MAPK and PI3K-AKT branches triggered by PDGF-BB was significantly suppressed upon Tudor-SN knockdown in MASMCs. Conversely, overexpression of Tudor-SN overactivated these signaling pathways, leading to facilitated proliferation and migration of MASMCs. Consequently, we aimed to determine the precise impact of Tudor-SN on these signaling pathways. GO analysis of the identified proteins that interact with Tudor-SN revealed that PTEN, a well-established tumor suppressor protein, is particularly intriguing. Previous studies have confirmed the pivotal role of PTEN in maintaining the contractile phenotype and vascular homeostasis of VSMCs. Given the fascinating results showing that Tudor-SN modulates PTEN protein levels, we hypothesized that PTEN might play a crucial role in mediating the regulatory effects of Tudor-SN on the vascular smooth muscle phenotype.

The multidomain composition of Tudor-SN is the primary factor contributing to its various biological functions. Specifically, the N-terminal SN domains act as a bridge between components in gene transcription and play vital roles in the formation of the RISC complex, while the C-terminal Tudor domain is believed to participate in RNA splicing [12]. For further elucidation of the interaction between Tudor-SN and PTEN, distinct domains of Tudor-SN were cloned into different plasmids, and subsequent Co-IP assays localized the binding site of PTEN within the SN1 domain. In support of this finding, the absence of the SN1 domain in the mutant Tudor-SN led to an absence of interaction with PTEN, revealing a potential functional role of the SN domain in maintaining vascular homeostasis.

The primary pathways involved in protein degradation in eukaryotic cells currently include the autophagy‒lysosome pathway and the ubiquitin‒proteasome pathway. The former primarily targets extracellular components as well as endocytic proteins [45], while the latter serves as the principal degradation route for most cellular proteins [46]. To date, limited research has been conducted on the relationship between Tudor-SN and protein degradation and the precise molecular mechanisms involved remain unclear.

NEDD4-1, a widely known E3 ubiquitin protein ligase of PTEN, has diverse effects on cardiovascular diseases, including cardioprotective effects following myocardial ischemia‒reperfusion injury [70] and regulation of vascular calcification by modulating bone formation signals [71]. Our present study revealed the novel bridging role of Tudor-SN in mediating the interaction between NEDD4-1 and PTEN, emphasizing that Tudor-SN exacerbates pathological intimal hyperplasia by facilitating NEDD4-1-dependent polyubiquitination of PTEN. Consistent with these findings, the effects of Tudor-SN deletion were attenuated by PTEN knockdown or inhibition. These findings provide a promising therapeutic target for preventing pathological vascular remodeling. In addition, while Tudor-SN and PTEN have been found in multiple types of tumor cells [72–76], their protein interactions were first discovered here and warrant extensive exploration regarding their ability to control tumorigenesis.

Additionally, the involvement of Tudor-SN in the process of atherosclerotic plaque formation was also investigated. A higher expression level of Tudor-SN was observed within the plaque of *Apoe*^*−/−*^ mice compared to the control group, and TSN^smcKO^*Apoe*^*−/−*^ mice exhibited a significant reduction in the overall plaque area throughout the entire aorta. The decreased necrosis area and increased collagen content indicated a protective role of Tudor-SN knockout. Notably, while PTEN has been established as playing a critical role in the atherosclerosis progression, further experiments are warranted to determine whether Tudor-SN acts upstream of PTEN during this process.

## Conclusion

In summary, our study revealed a notable role of Tudor-SN as a regulator during pathological vascular remodeling in a PTEN-dependent manner. Through mediating the interaction between NEDD4-1 and PTEN, Tudor-SN facilitates PTEN polyubiquitination and activates the downstream signaling pathways. The deletion of Tudor-SN in VSMCs effectively attenuates the synthetic phenotypic switching and vascular remodeling under pathological conditions. Together, these findings suggest Tudor-SN as a potetial new therapeutic target for preventing vascular remodeling diseases.

## Supplementary Information

Supplementary material 1.

## Data Availability

The data underlying this article will be shared on reasonable request to the corresponding author.
