# Supplementary material for: Tudor-SN exacerbates pathological vascular remodeling by promoting the polyubiquitination of PTEN via NEDD4-1
Source: J Biomed Sci. 2024 Sep 5;31:88. doi: 10.1186/s12929-024-01076-9 (PMC11378411; doi:10.1186/s12929-024-01076-9)
Supplement: Supplementary file 1 — Supplementary material 1. [file 12929_2024_1076_MOESM1_ESM.docx]

**Supplemental Files**


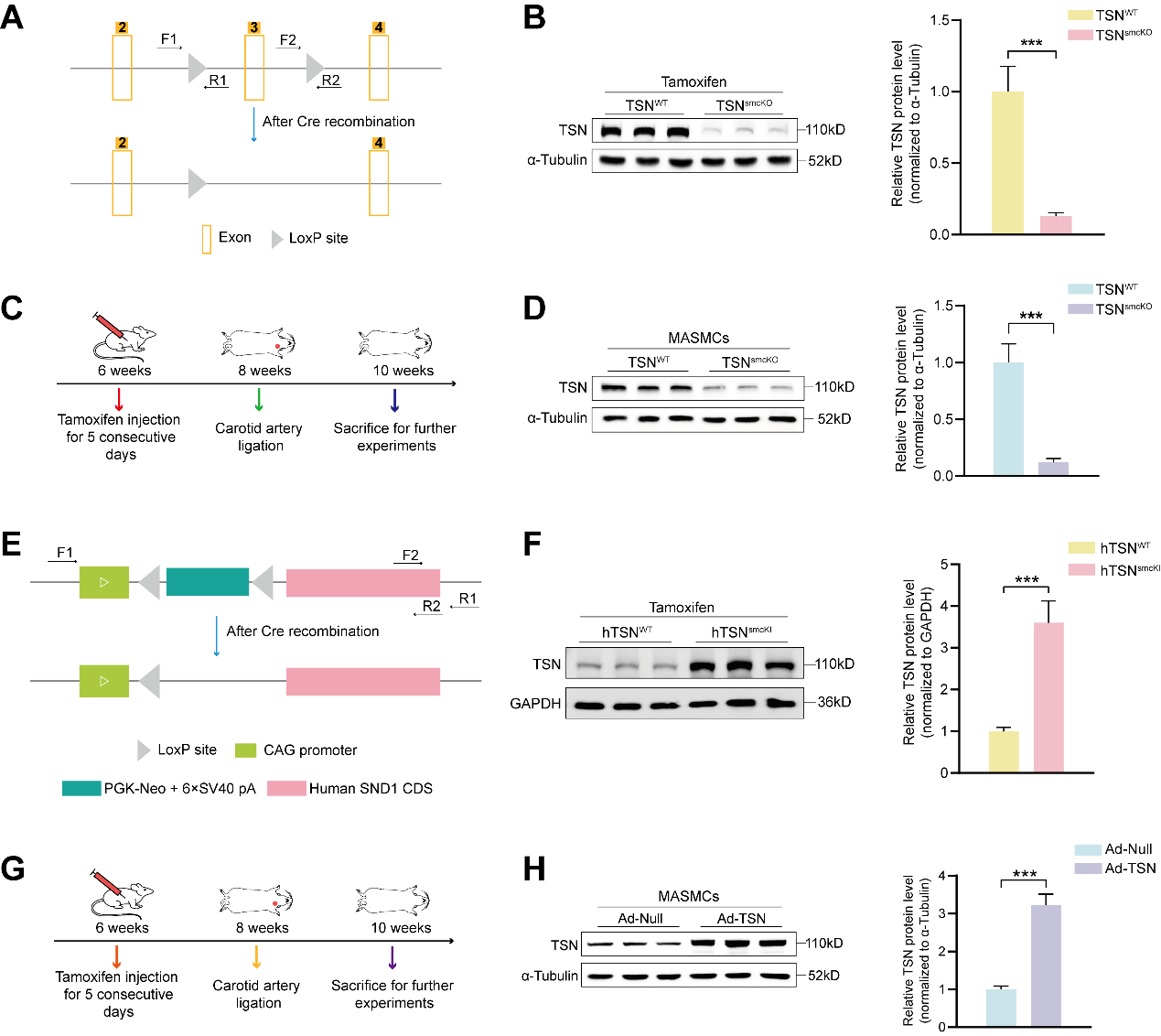


**Supplemental Figure 1. Verification of Tudor-SN deletion and overexpression in vascular smooth muscle cells.** (**A**) The design of the TSN^smcKO^ mice. (**B,D**) Tudor-SN deletion was verified in mouse aortas (B) and primary MASMCs (D). (**C**) Schematic illustration of the experimental procedures. (**E**) The design of the hTSN^smcKI^ mice. (**F,H**) Tudor-SN overexpression was verified in mouse aortas (F) and primary MASMCs (H). (**G**) Schematic illustration of the experimental procedures. The results are representative of at least three independent experiments and are shown as the mean ± SD; statistical analyses were conducted using Student’s t tests; ***p < 0.001.


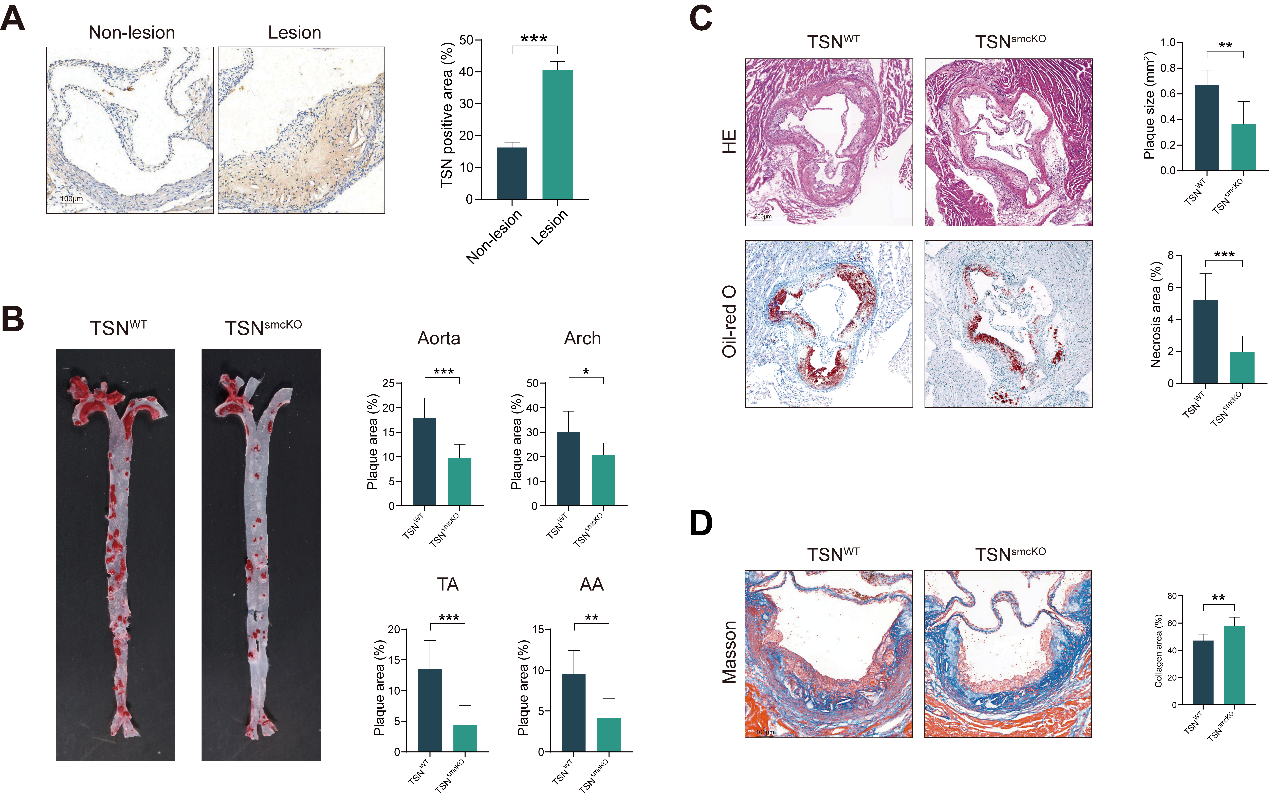


**Supplemental Figure 2. Tudor-SN deficiency alleviates the progression of atherosclerosis in hyperlipidemic *Apoe^-/-^* mice. (A)** Immunohistochemistry of Tudor-SN within the atherosclerotic plaque of *Apoe^-/-^* mice fed with a high-fat diet for 16 weeks. Scale bar: 100 μm. The percentage of Tudor-SN positive area was analyzed on the right. **(B)** Representative images of en face Oil Red O staining of whole aortas from TSN^WT^*Apoe^-/-^* and TSN^smcKO^*Apoe^-/-^* mice (n=8). The statistical analyses of the plaque area are shown on the right. TA: thoracic aorta; AA: abdominal aorta. **(C)** H&E and Oil red O staining of the aortic root from TSN^WT^*Apoe^-/-^* and TSN^smcKO^*Apoe^-/-^* mice (n=8). Scale bar: 200 μm. The statistical analyses of plaque size and the percentage of necrosis area are shown on the right. **(D)** Masson staining and quantification of collagen area of the aortic root from TSN^WT^*Apoe^-/-^* and TSN^smcKO^*Apoe^-/-^* mice (n=8). Scale bar: 100 μm. Throughout, the results are presented as the mean ± SD; statistical analyses were conducted using Student’s t tests; *p < 0.05, **p < 0.01, ***p < 0.001.


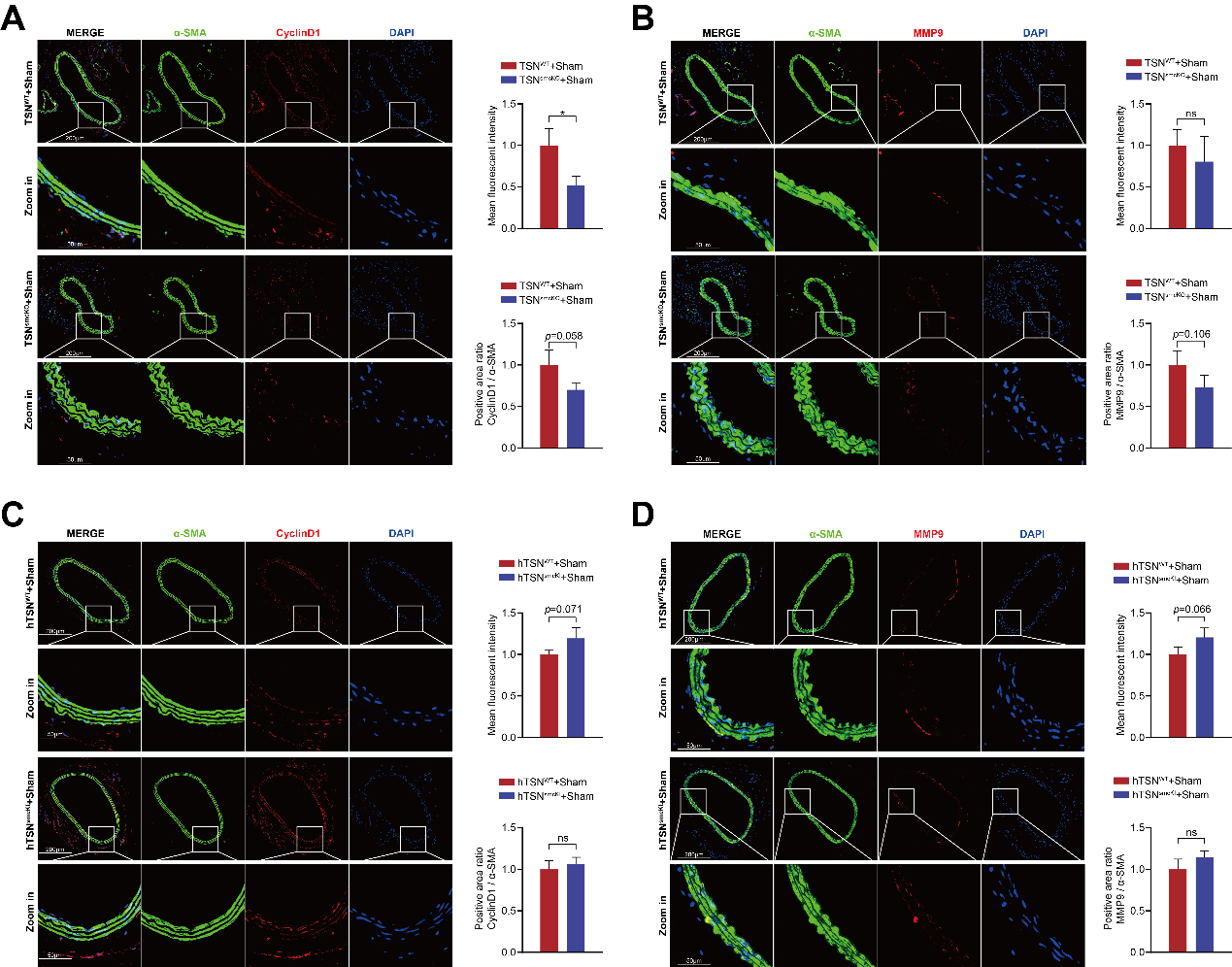


**Supplemental Figure 3. The effects of Tudor-SN deletion and overexpression on sham-operated arteries.** (**A,B**) Double immunofluorescence staining of α-SMA (green), CyclinD1 (A, red) and MMP9 (B, red) in sham-operated artery sections from TSN^WT^ and TSN^smcKO^ mice 14 days postsurgery (n=3). Nuclei were stained with DAPI (blue). Scale bar: 200 μm and 50 μm (zoom in). Statistical analyses of the mean fluorescence intensity and positive area ratio of CyclinD1/α-SMA (A) or MMP9/α-SMA (B) are shown on the right (both were normalized to those of the TSN^WT^ group). (**C,D**) Double immunofluorescence staining of α-SMA (green), CyclinD1 (C, red) and MMP9 (D, red) in sham-operated artery sections from hTSN^WT^ and hTSN^smcKI^ mice 14 days postsurgery (n=3). Nuclei were stained with DAPI (blue). Scale bar: 200 μm and 50 μm (zoom in). Statistical analyses of the mean fluorescence intensity and percentage of cells positive for CyclinD1/α-SMA (C) or MMP9/α-SMA (D) are shown on the right (both were normalized to those in the hTSN^WT^ group). Throughout, the results are presented as the mean ± SD; statistical analyses were conducted using Student’s t tests; *p < 0.05; ns, nonsignificant.


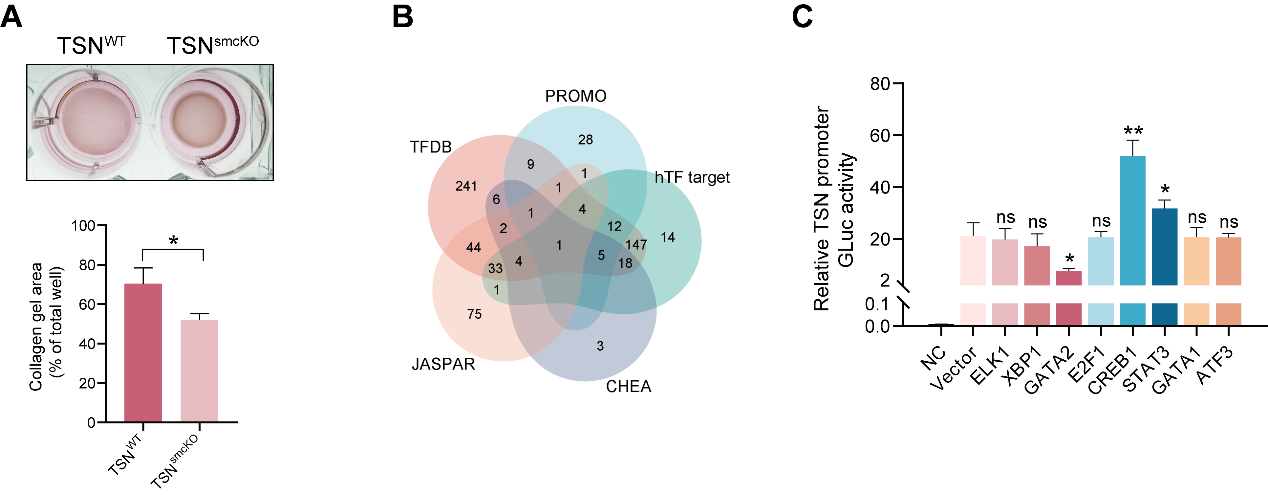


**Supplemental Figure 4. (A)** MASMCs isolated from TSN^WT^ and TSN^smcKO^ mice were harvested and subjected to collagen gel contraction assay (n=3). The photos were taken 24 hours after collagen polymerization, and the size of the collagen gel in each well was evaluated. **(B)** Venn diagram showing putative upstream transcription factors of Tudor-SN predicted by AnimalTFDB, PROMO, hTF target, JASPAR and CHEA. **(C)** Luciferase reporter assays of several interested transcription factors (n=3). Throughout, the results are presented as the mean ± SD; statistical analyses were conducted using Student’s t tests; *p < 0.05, **p < 0.01; ns, nonsignificant.


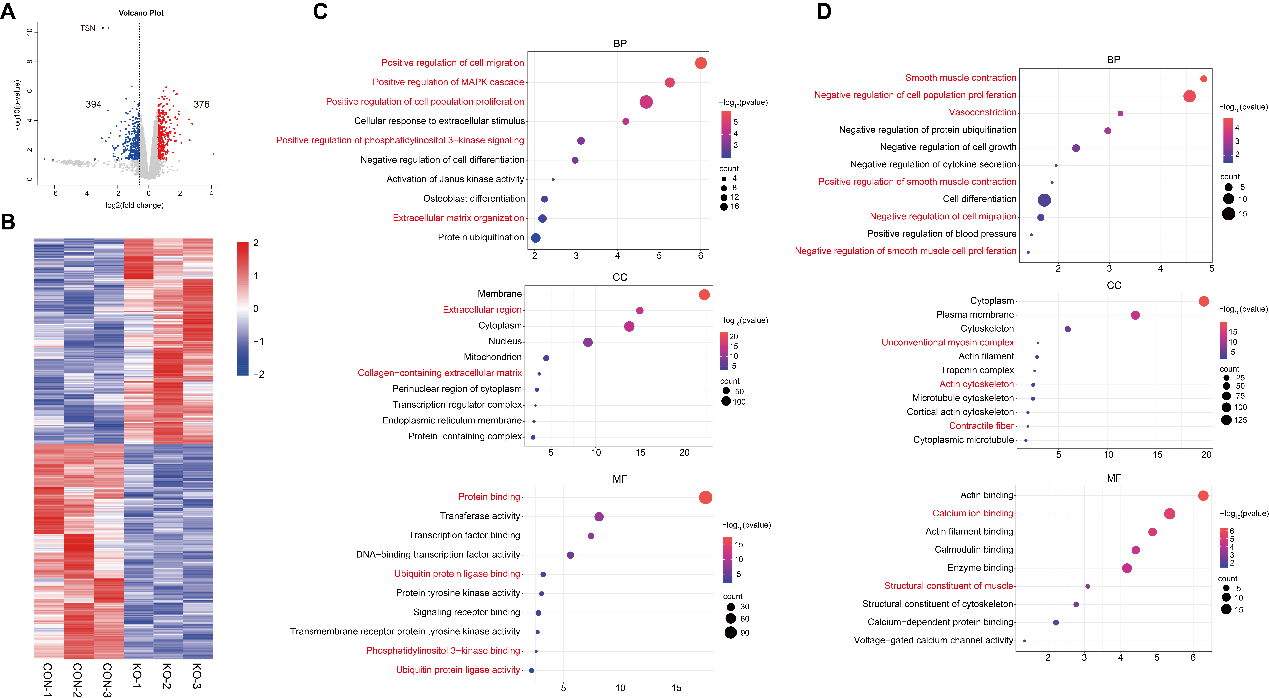


**Supplemental Figure 5. Additional transcriptome sequencing results.** (**A**) Volcano plot depicting the genes differentially expressed between TSN^WT^ and TSN^smcKO^ aortas (n=3). (**B**) Heatmap of all DEGs in aortas from TSN^WT^ (CON) and TSN^smcKO^ (KO) mice (n=3). (**C,D**) GO enrichment analysis of downregulated genes (C) and upregulated genes (D) in TSN^smcKO^ mice. The terms of interest are marked in red.

**Supplementary Table S1**

| **Primer** | **Sequence（5’-3’）** |
| --- | --- |
| m-18S-F | GCACCACCACCCACGGAATCG |
| m-18S-R | TTGACGGAAGGGCACCACCAG |
| m-TSN-F | CTGCTGGCCGGCATTGA |
| m-TSN-R | AGGCATGCCACATAGAGCAA |
| h-TSN-F | CTCCAAGGTCCACTTCACCG |
| h-TSN-R | TAGTGGGCCCAGACCTTCTC |
| m-P53-F | ATTCAGGCCCTCATCCTCCT |
| m-P53-R | CCATGGCAGTCATCCAGTCT |
| m-CREBBP-F | GGAAAGCCTGCCAAGTTGC |
| m-CREBBP-R | ACAGTCATGTCGTGTGCAGT |
| m-PTEN-F | GCCAAGTCCAGAGCCATTTC |
| m-PTEN-R | TGCTTTGAATCCAAAAACCTTACT |
| h-TSN promoter-F | CCCCTTCCCAACTCCCCTTGA |
| h-TSN promoter-R | ACGCGATCTCCGCCGC |

Table S1. The primer sequence of the target genes.
